# TRB proteins in moss reveal their evolutionarily conserved roles in plant development and telomere maintenance

**DOI:** 10.1111/tpj.70574

**Published:** 2025-11-14

**Authors:** Alžbeta Kusová, Marcela Holá, Ivana Goffová Petrová, Jiří Rudolf, Dagmar Zachová, Jan Skalák, Jan Hejátko, Božena Klodová, Tereza Přerovská, Martin Lyčka, Eva Sýkorová, Yann J. K. Bertrand, Jiří Fajkus, David Honys, Petra Procházková Schrumpfová

**Affiliations:** ^1^ Laboratory of Functional Genomics and Proteomics, National Centre for Biomolecular Research, Faculty of Science Masaryk University Brno Czech Republic; ^2^ Mendel Centre for Plant Genomics and Proteomics, Central European Institute of Technology Masaryk University Brno Czech Republic; ^3^ Institute of Experimental Botany of the Czech Academy of Sciences Prague Czech Republic; ^4^ Institute of Biophysics of the Czech Academy of Sciences Brno Czech Republic; ^5^ Institute of Botany of the Czech Academy of Sciences Průhonice Czech Republic

**Keywords:** TRB, telomeres, gene regulation, protonema, mosses, plant evolution

## Abstract

Telomere repeat binding (TRB) proteins are plant‐specific proteins with a unique domain structure distinct from telomerebinding proteins in animals and yeast. While extensively studied in seed plants, their role in early‐diverging plant lineages remains largely unexplored. Here, we investigate TRB proteins in a model moss, *Physcomitrium patens*, to assess their evolutionary conservation and functional significance. Functional analysis using single knockout mutants revealed that individual *PpTRB* genes are essential for normal development, with mutants exhibiting defects in the two‐dimensional (protonemal) stage, and more prominently, in the formation of three‐dimensional (gametophore) structures. Some double mutants displayed telomere shortening, a phenotype also observed in TRB‐deficient seed plants, indicating a conserved role for TRBs in telomere maintenance. Transcriptome profiling of TRB mutants revealed altered expression of genes associated with transcriptional regulation and stimulus response in protonema. Subcellular localization studies across various plant cell types confirmed that PpTRBs, like their seed plant counterparts, localize prevalently to the plant nucleus and mutually interact. In bryophytes, TRBs form a monophyletic group that mirrors the species phylogeny, whereas in seed plants, TRBs have diversified into two distinct monophyletic groups. Our findings provide the first comprehensive characterization of TRB proteins in non‐vascular plants and demonstrate their conserved roles in telomere maintenance, with additional implications for plant development and gene regulation across land plant lineages.

## Significance Statement

TRB proteins in the moss *P. patens* are essential for normal development, telomere maintenance, and transcriptional regulation. Our findings demonstrate the evolutionary conservation of TRB functions across land plants and highlight their ancient origin in streptophytes.

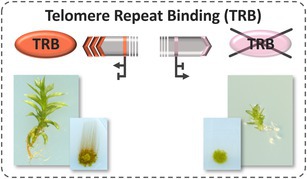

Telomere repeat binding (TRB) proteins regulate telomere maintenance and gene expression in seed plants, but their roles in early‐diverging plants remain unknown. This study presents the first functional characterization of TRBs in the moss *Physcomitrium patens*, showing they are essential for growth, telomere integrity, and transcriptional regulation. Genetic, transcriptomic, and phylogenetic analyses reveal TRB conservation across land plants and suggest their origin in streptophyte algae, offering insights into the evolution of plant‐specific regulatory pathways.

## INTRODUCTION

Telomere repeat binding (TRB) proteins were initially characterized in seed plants for their roles in telomere maintenance (Marian et al., 2003; Schrumpfová et al., 2004, 2008) as well as in gene regulation (Kusová et al., 2023; Schrumpfová et al., 2016; Tan et al., 2018; Wang et al., 2023; Zhou et al., 2018). TRB proteins exhibit a plant‐specific domain architecture comprising an N‐terminal Myb‐like domain, a histone‐like (H1/5‐like) domain, and a coiled‐coil domain at the C‐terminus. All five members of the TRB protein family in *Arabidopsis thaliana* have been shown to bind plant telomeric repeats (TTTAGGG)_n_ through their Myb‐like domain (Kusová et al., 2023; Mozgová et al., 2008). The centrally positioned H1/5‐like domain mediates protein–protein interactions, as demonstrated in our previous studies (Kuchar & Fajkus, 2004; Schrumpfová et al., 2008).

TRB proteins contribute to telomere maintenance, as demonstrated by telomere length variation in *A. thaliana* mutants in some *AtTRB* genes (Amiard et al., 2024; Schrumpfová et al., 2014; Zhou et al., 2018). All TRB family members in *A. thaliana* are proposed to participate in the biogenesis of the enzyme responsible for maintaining telomere length, named telomerase. TRBs interact directly with the catalytic protein subunit of telomerase (Kusová et al., 2023; Schrumpfová et al., 2014) and with plant homologs of key components of the mammalian telomere complex Shelterin, which is responsible for maintaining telomere sequences (e.g., AtPot1a/b; Protection of Telomeres 1a/b) (Kuchar & Fajkus, 2004; Kusová et al., 2023; Schrumpfová et al., 2008). In line with that, *in situ* co‐localization of AtTRB1 with long telomeric DNA repeats was observed in plant cells (Dreissig et al., 2017; Schrumpfová et al., 2014).

The Myb‐like domain of TRB proteins shares structural similarity with the Myb‐like domain of human Telomeric repeat‐binding factors 1 and 2 (hTRF1 and hTRF2) (Broccoli et al., 1997), which are the components of the Shelterin complex, responsible for mediating the direct binding of the complex to telomeric DNA sequences. However, unlike TRB proteins, hTRFs possess the Myb‐like domain at the C‐terminus and lack H1/5‐like and coiled‐coil domains. Surprisingly, in *Arabidopsis*, similarly structured proteins as human hTRFs, named TRF‐Like proteins (TRFLs), possess a C‐terminal Myb domain but do not participate in telomere maintenance (Fulcher & Riha, 2016) and require an accessory Myb‐extension domain for binding telomeric dsDNA *in vitro* (Karamysheva et al., 2004; Ko et al., 2008).

The function of TRB proteins extends beyond their association with telomeres and telomerase. In addition to binding long‐terminal telomeric repeats, TRB proteins also interact with other DNA sequences, positioned interstitially, as are short interstitial telomeric sequences, known as *telo*‐box motifs (Regad et al., 1994; Schrumpfová et al., 2016), as well as to Site II motifs (Wang et al., 2023; Zhou et al., 2016), JMJ14 binding motifs (Wang et al., 2023) and other motifs which are present in the promoter regions of numerous genes. The binding of AtTRBs to *telo*‐boxes facilitates the recruitment of various protein complexes, such as JMJ14 (Jumonji domain‐containing protein 14), PRC2 (Polycomb‐Repressive Complex 2), or PEAT complex (PWOs‐EPCRs‐ARIDs‐TRBs). These complexes, recruited by TRBs, have diverse roles in regulating gene expression, including histone H3K4 demethylation (Amiard et al., 2024; Wang et al., 2023) and histone H3K27 methylation (Kusová et al., 2023; Xuan et al., 2024; Zhou et al., 2018), as well as histone H2A deubiquitination and histone H4 acetylation, which may contribute to heterochromatin silencing (Tan et al., 2018; Tsuzuki & Wierzbicki, 2018) as well as the formation of an active chromatin state (Zheng et al., 2023).

In our previous phylogenetic analysis, we reported evidence of variable rates of TRB gene duplication and copy retention in Streptophyta genomes (Kusová et al., 2023). We suggested that TRB proteins first evolved within *Klebsormidiophyceae*; however, the occurrence of TRBs in many early‐diverging streptophytes remained unresolved due to the limited genomic data. In Bryophyta, we identified TRB genes with very high pairwise sequence similarity to each other.

The genomes of spermatophytes (seed plants) have been shaped by several rounds of whole‐genome duplication events (WGDs) not shared with Bryophyta and Lycophyta (Clark & Donoghue, 2018). The history of WGD is reflected in the TRB phylogeny, as two main distinct lineages in seed plants can be recognized (Kusová et al., 2023; Marian et al., 2003; Zhou et al., 2016). In *A. thaliana*, these two TRB lineages are represented by three paralogs (AtTRB1–3), and two additional paralogs (AtTRB4–5), respectively (Kusová et al., 2023; Schrumpfová et al., 2014).

In the spreading earth moss *Physcomitrium patens* (formerly *Physcomitrella*), three proteins with high sequence similarity, containing a unique Bryophyte‐specific motif, were identified through our *in silico* analysis (Kusová et al., 2023). The moss *P. patens* was the first bryophyte to have its genome sequenced (Lang et al., 2018; Rensing et al., 2008) and may serve as a model system to investigate the shift from the two‐dimensional filamentous phase of the life cycle (protonemata, composed of chloronemal and caulonemal cells) to the three‐dimensional (gametophores) stages (de Keijzer et al., 2021; Fernandez‐Pozo et al., 2020; Moody, 2022; Prigge & Bezanilla, 2010; Rensing et al., 2020; Roberts et al., 2012; Strotbek et al., 2013). As in other mosses, the dominance of the haploid phase during the *P. patens* life cycle simplifies genetic manipulations (Prigge & Bezanilla, 2010), and its highly efficient system of homologous recombination (HR) offers a straightforward approach for targeted gene replacement (Schaefer, 2002; Schaefer & Zrÿd, 1997). Altogether, *P. patens* is an excellent system for answering questions in evolutionary developmental biology (reviewed in Rensing et al., 2020), including whether TRBs retain their roles and functions, as characterized in *A. thaliana*, in early‐diverging plants.

In this study, we conduct a comprehensive characterization of three TRB proteins from the moss *P. patens* (PpTRB1, PpTRB2, and PpTRB3). Phenotypic analysis of various single and double‐*pptrb* knockout/knockdown mutants displays pronounced morphological growth defects during both the filamentous stages and leafy shoot‐like structures (protonemata and gametophore, respectively). Notably, double mutants *pptrb1 pptrb3* and *pptrb2 pptrb3* exhibit telomere shortening which remains consistent across subsequent generations. Transcriptomic analysis of the protonemal stage reveals alterations in the expression of genes encoding proteins involved in DNA transcription regulation, as well as those associated with stimulus response and cellular membrane and peripheral structures. Localization studies in diverse plant cell types demonstrate that PpTRB proteins are predominantly targeted to the nucleus and/or nucleolus. Additionally, mutual interactions between PpTRB proteins were observed in nuclear foci, as well as in the cytoplasm and at the cellular periphery. A comprehensive phylogenetic analysis reveals the presence of *TRB* genes throughout the plant phylogeny, including in streptophyte algae, hornworts, liverworts, mosses, and seed plants. Collectively, our findings provide the first detailed characterization of TRB proteins in mosses, demonstrating their roles in regulating plant growth, maintaining telomere homeostasis, and modulating gene expression. These results highlight the evolutionary conservation of TRB protein functions across early‐diverging plant lineages.

## RESULTS

### Mutant *pptrb* lines exhibit developmental defects

Our *in silico* analysis previously identified three TRB genes in *P. patens*, each containing the Myb‐like, H1/5‐like, and coiled‐coil domains, plus a bryophyte‐specific motif between the latter two (Kusová et al., 2023). In *A. thaliana*, TRBs are functionally redundant, with developmental defects arising only when multiple family members are mutated (Amiard et al., 2024; Wang et al., 2023; Zhou et al., 2018).

To investigate the role of PpTRBs in moss viability, we created various single‐ and double‐mutant lines (Figures S1 and S2). The *pptrb1* mutant line was generated by introducing a stop codon into the sequence coding for the Myb‐like domain using CRISPR/Cas9‐mediated homology‐directed repair, resulting in a knockdown mutant line. This was confirmed by RNA‐seq QuantSeq which targets the 3′ end of RNA transcripts (details on QuantSeq are provided below; Figure S3). The knockout mutants of *pptrb2* and *pptrb3* were generated using well‐established HR protocols (Kamisugi et al., 2006; Schaefer & Zrÿd, 1997), and also confirmed by QuantSeq. Double‐mutant lines (*pptrb1 pptrb2*, *pptrb1 pptrb3*, *pptrb2 pptrb3*) were generated from single mutants as described in the Methods section. Despite multiple attempts, we were unable to generate a single *pptrb1* mutant using HR procedures, nor were we able to generate a triple *pptrb1‐3* mutant line. Between two and four independently generated mutant lines for each single or double‐mutant genotype were selected for further analysis (Figure S2).

**Figure 1:**
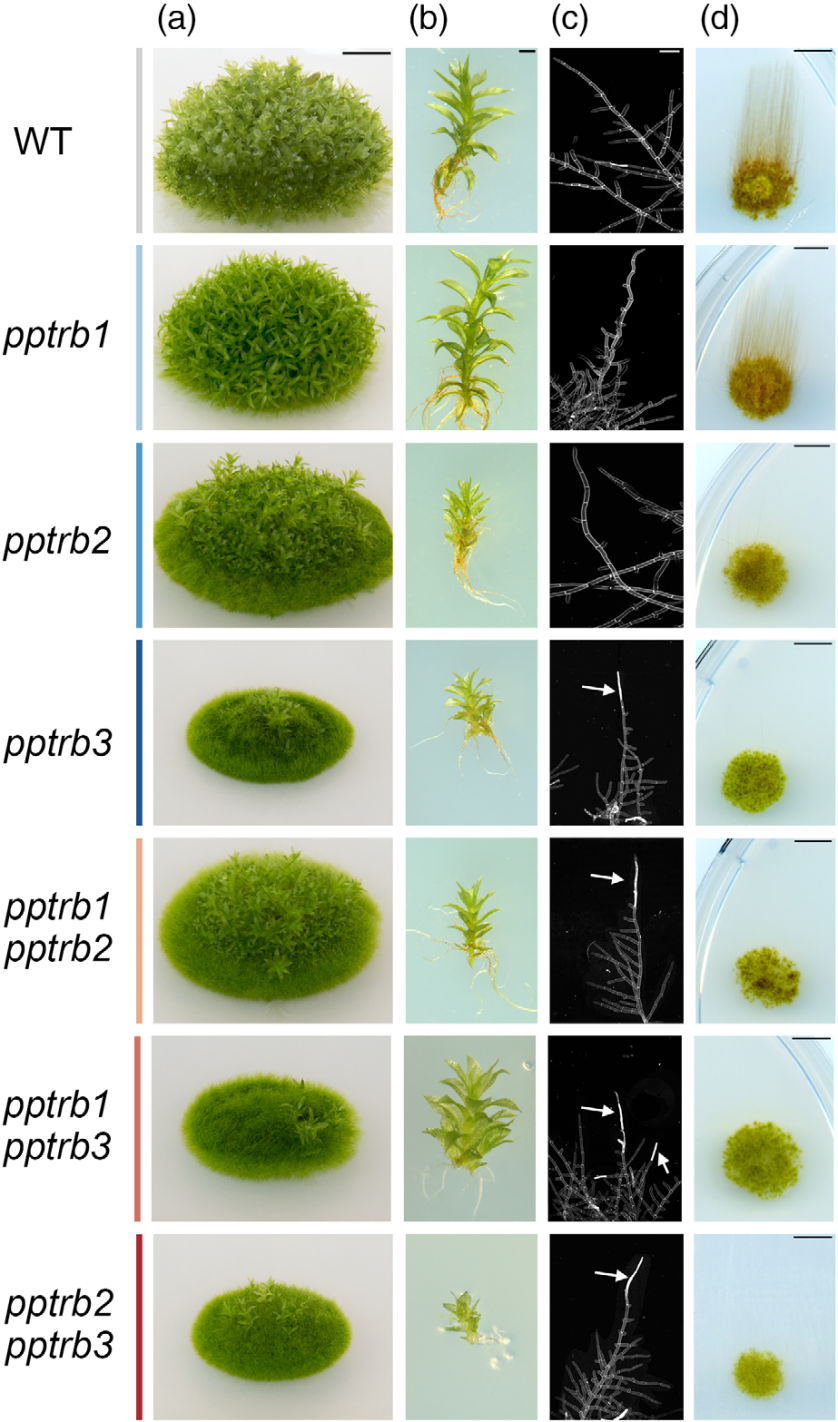
The *pptrb* mutant lines exhibited reduced growth of protonema and, particularly, gametophores. (a) Representative morphotypes of 1‐month‐old wild‐type (WT) and mutant lines grown on BCDAT medium. Scale bar = 5 mm. (b) Close‐up view of 1‐month‐old gametophores of WT and mutant lines. Scale bar = 1 mm. (c) Propidium iodide staining of 10‐day‐old protonema grown on BCDAT medium showing defective apical caulonemal cells that are indicated by arrows in *pptrb3*, *pptrb1 pptrb2*, *pptrb1 pptrb3*, and *pptrb2 pptrb3* mutant lines. Scale bar = 100 μm. (d) Impaired caulonema formation after 3 weeks in dark conditions. Scale bar = 5 mm.

The *pptrb2* and *pptrb3* single mutants, as well as all double‐*pptrb* mutants, exhibited delayed development of macroscopic gametophores compared with the wild‐type (WT) moss (Figure 1a,b; Figure S2). Notably, the double mutants *pptrb1 pptrb3* and *pptrb2 pptrb3* displayed a predominance of protonemal stages over gametophores. Additionally, propidium iodide (PI) staining of 10‐day‐old protonema revealed that most of the apical caulonemal cells (positioned at the growing tip) in mutant lines were defective, presenting premature senescence and progressing to cell death (Figure 1c).

The transition from chloronema to caulonema in the mutant lines was investigated by exploiting the ability of *P. patens* to develop caulonemata, but not chloronemata, under dark conditions (Cove et al., 1978). Protonema grown for 1 week was transferred—either as homogenized or unhomogenized samples—to new plates, grown for an additional week in the light, and then placed in the dark for 3 weeks. Under these conditions, WT plants developed long, negatively gravitropic caulonemata, whereas *pptrb2*, *pptrb3*, and all double mutants displayed significantly impaired caulonemal growth (Figure 1d; Figure S4). This impaired caulonemal formation likely played a critical role in the defects observed during gametophore development, as caulonemata are essential for transitioning to the differentiation of cells that eventually form gametophores (Cove & Knight, 1993; Thelander et al., 2018).

Overall, the defects in protonema growth, caulonema development, and gametophore formation detected in the *pptrb* mutants suggest that TRB proteins play a critical role in the early stages of moss development. Specifically, these proteins appear to regulate the transition from the protonemal stage to gametophore formation in *P. patens*. Furthermore, the inability to generate triple *pptrb* mutant lines indicates that PpTRB proteins are likely involved in essential pathways necessary for moss viability and development.

### Role of PpTRB proteins in transcriptional regulation

Since AtTRB proteins are known to be involved in gene regulation in *Arabidopsis* (Schrumpfová et al., 2016; Wang et al., 2023; Zhou et al., 2018), we sought to determine whether they retain this role in *P. patens* as well. To identify genes differentially regulated by PpTRBs, we performed QuantSeq targeting the 3′ end of the polyadenylated RNA isolated from 7‐day‐old protonema from single‐ and double‐*pptrb* mutants and compared the results with WT plants.

**Figure 2:**
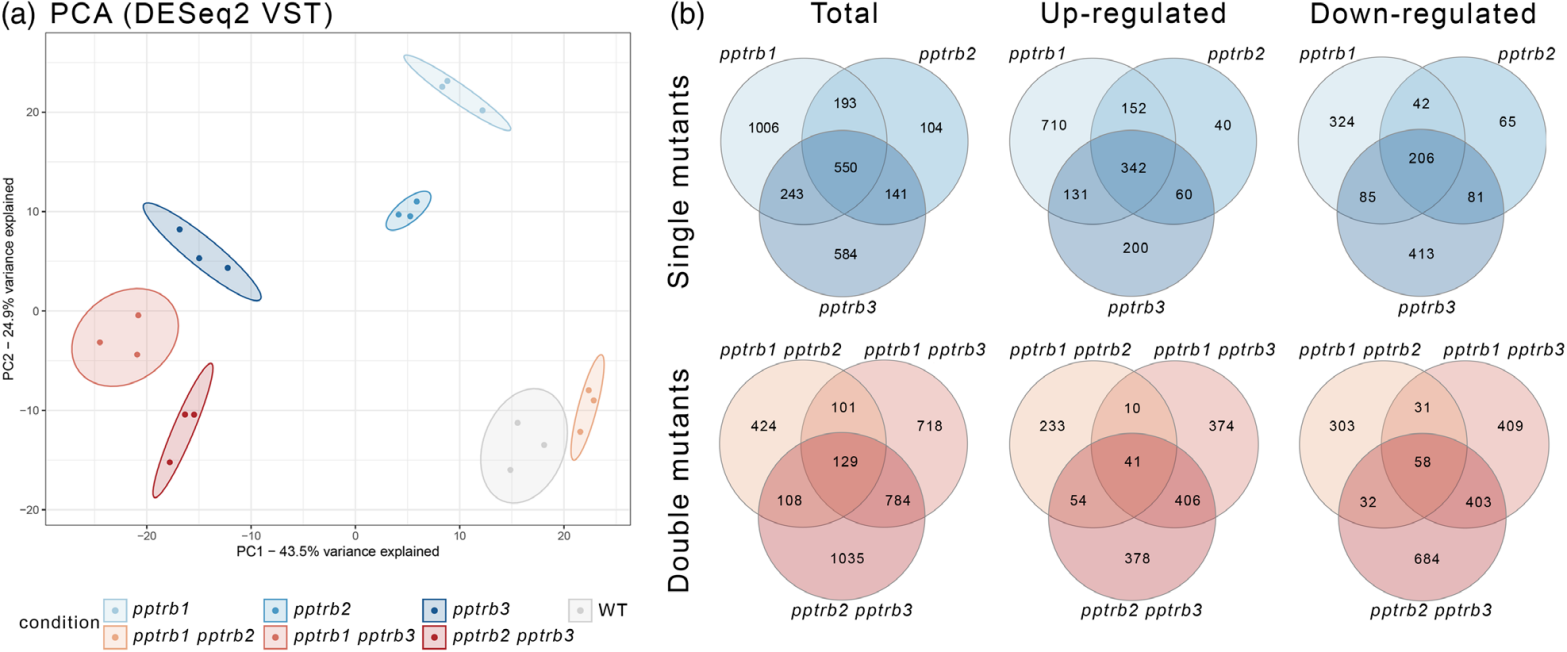
Differential gene expression in *pptrb* mutants. (a) Principal component analysis based on DESeq2 with variance stabilizing transformation reveals transcriptomic differences among *pptrb* single and double mutants when using the reference genome v3.3. Each mutant line is represented by a distinct color. (b) Venn diagram illustrating the overlap of differentially expressed genes (DEGs) among *pptrb* single and double mutants. The total number of DEGs, as well as the subsets of upregulated and downregulated genes, are indicated.

QuantSeq analysis revealed distinct differences in gene expression profiles between individual single‐ and double‐*pptrb* mutants, analyzed across three biological replicates. Notably, mutants with *pptrb3* (either single or double), are clearly separated from other genotypes in the principal component analysis (PCA) plot (Figure 2a). Numerous differentially expressed genes (DEGs), both upregulated and downregulated, were identified in *pptrb* mutant plants compared with the WT when mapped against Ppatens_318_v3.3 (v3.3) and Ppatens_870_v6.1 genomes (v6.1) (Table S1).

Overlapping DEGs among the mutants that were significantly up‐ or downregulated are depicted in Venn diagrams. A total of 550 and 129 DEGs were found to overlap among single‐ and double‐mutant datasets, respectively, using v3.3 genome (Figure 2b) and 547 and 133 DEGs were found using v6.1 genome (Figure S5). Interestingly, the overlap between *pptrb1 pptrb3* and *pptrb2 pptrb3* was higher than the overlap between *pptrb1 pptrb3* and *pptrb1 pptrb2* (Figures S6 and S7).

**Figure 3:**
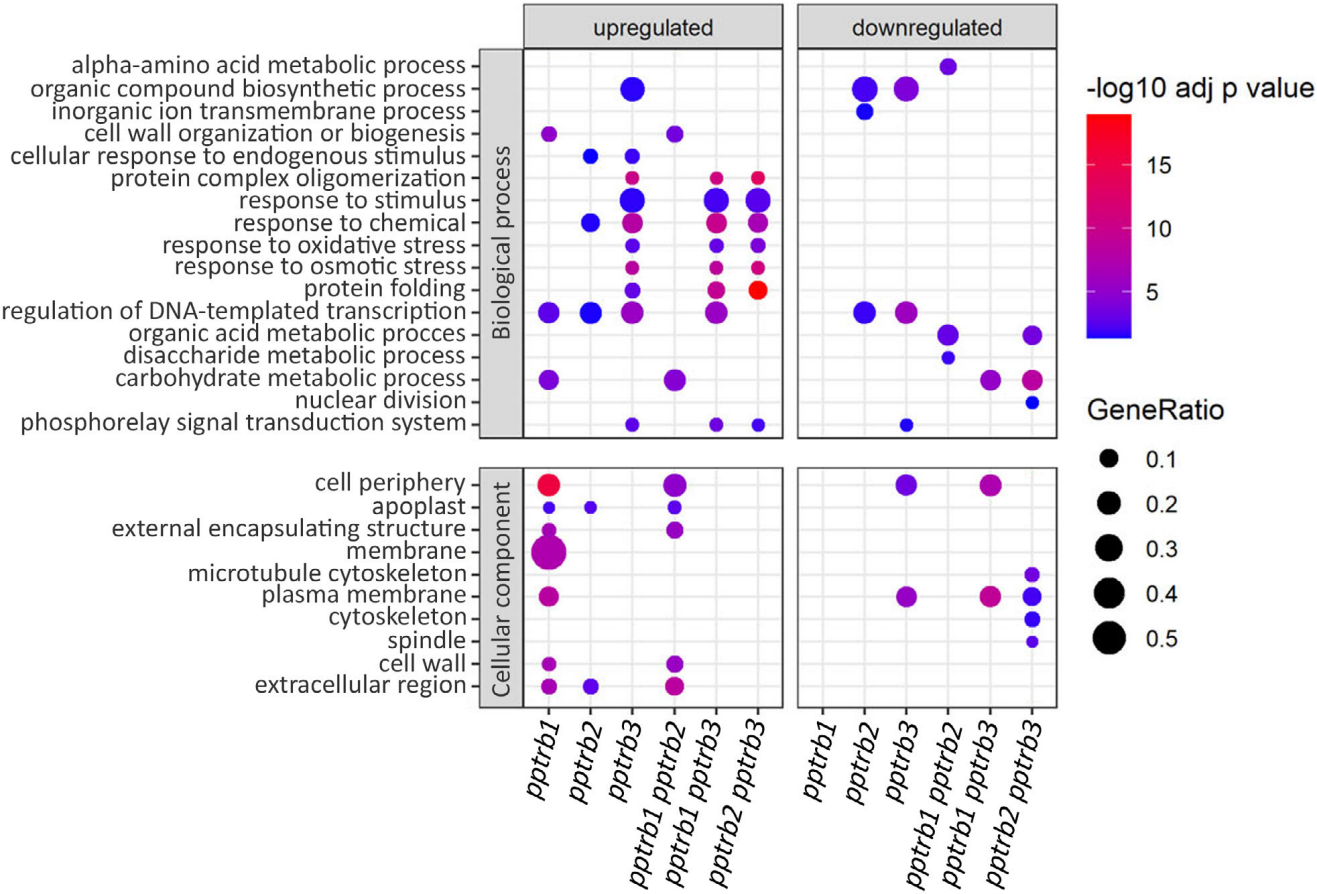
Gene ontology (GO) enrichment analysis of differentially expressed genes in *pptrb* mutant lines. GO enrichment analysis was performed separately for upregulated and downregulated genes identified in *pptrb* mutants relative to wild‐type. Among the upregulated differentially expressed genes (DEGs), GO terms significantly enriched in the Biological Process (GO:BP) category included ‘regulation of DNA‐templated transcription’ and ‘response to stimuli’. Within the Cellular Component (GO:CC) category, enriched terms were associated with ‘cell periphery’, ‘membrane’, ‘cell wall’, and ‘extracellular region’. In downregulated DEGs, GO:BP enrichment showed ‘compound biosynthetic processes’ and again in ‘regulation of DNA‐templated transcription’. GO:CC terms for these genes were similarly enriched in ‘cell periphery’ and ‘plasma membrane’. Adjusted *P*‐values for each GO term were derived from g:Profiler. GeneRatio—the ratio of DEGs annotated to the GO term calculated by dividing intersection_size by query_size from the g:Profiler. For full GO enrichment analysis, see Figure S8.

Gene ontology (GO) analysis of RNA‐seq data for upregulated DEGs revealed significant enrichment in GO Biological Process (GO:BP) terms ‘regulation of DNA‐templated transcription’and ‘response to stimuli’. In GO Cellular Components (GO:CC), enriched terms included ‘cell periphery’, ‘membrane’, ‘cell wall’ and ‘extracellular region’, For downregulated DEGs, GO:BP analysis highlighted terms related to ‘compound of biosynthetic processes’ and ‘regulation of DNA‐templated transcription’. The GO:CC analysis of the downregulated DEGs showed enrichment in ‘cell periphery’ and ‘plasma membrane’ terms (Figure 3; Figure S8).

We utilized our custom program GOLEM (Gene RegulatOry ELeMents) (Nevosád et al., 2025), to analyze the frequency and distribution of *telo*‐box motifs, a key recognition motif for TRB proteins within gene promoters relative to the transcription start site (TSS). The analysis was conducted on genes with the highest transcription levels (20th percentile) in single and double‐*pptrb* mutant lines, (Table S2a), comparing them to both the genome‐wide distribution and the frequency in WT plants. The *telo*‐box motif exhibits non‐random distribution with a higher occurrence upstream TSS (interval [−50; 0] relative to TSS; *P*‐value <10^−5^) (Gaspin et al., 2010; Liboz et al., 1990; Schrumpfová et al., 2016) (Figure S9). The relative peak value (number of motifs relative to number of genes involved in the defined percentile) is significantly higher in all the involved single‐ and double‐*pptrb* mutants but not in WT compared with the whole‐genome (Fisher's exact test; Bonferroni corrected *P*‐values <0.05) which indicates higher representation of genes with upstream TSS *telo*‐box motif in highly expressed genes in *pptrb* mutants. Comparing the mutants with WT, the single mutants *pptrb1, pptrb3*, and the double mutants *pptrb1 pptrb3 and pptrb2 pptrb3* exhibit significantly higher occurrence of *telo*‐box motifs in the upstream TSS peak in the promoters of highly expressed genes.

Genes containing a *telo*‐box motif near their TSS accounted for approximately 15–27% of the most highly transcribed genes in *pptrb* mutant lines but only 9% in WT plants. Additionally, in contrast to the more dispersed genome‐wide distribution, which reflects motif presence regardless of gene transcription, the *telo*‐box motif was clearly localized upstream of the TSS in genes highly transcribed in protonema. Interestingly, JMJ14 and Site II motifs do not show such strong motif enrichment compared with WT although they are functionally linked to AtTRB proteins (Wang et al., 2023; Zhou et al., 2018). In the case of the Site II motif, there is a significantly higher proportion of motifs in the upstream TSS peak compared with the whole genome only in the double‐mutant *pptrb1 pptrb3* (Fisher's exact test; Bonferroni corrected *P*‐value = 0.03), which indicates slightly higher representation of genes with upstream TSS Site II motif in highly expressed genes in *pptrb1 pptrb3*.

To further explore these genes, we performed GO analysis on the targets identified by the GOLEM program, that exhibited both the highest transcription levels (20th percentile) in *pptrb* mutant plants and the presence of a *telo*‐box in their promoters (±100 bp from the TSS). This analysis revealed a higher number of genes coding for ribosomal proteins in the mutant lines compared with the WT (Table S2b). This finding is consistent with previous observations of Schrumpfová et al. (2016), who demonstrated via ChIP‐Seq that AtTRB1 binds to *telo*‐box motifs in the promoters of genes associated with translation machinery, particularly ribosomal protein genes. Interestingly, when we analyzed only the promoters of DEGs, irrespective of their transcription levels, no significant enrichment of motifs or prevalence of ribosomal genes was observed.

Altogether, our RNA‐seq analysis of *pptrb* single and double mutants revealed distinct expression profiles, particularly in mutants involving *pptrb3*. GO analysis suggested that TRBs affect the expression of the genes associated with transcriptional regulation, response to various stimuli and cellular periphery functions. Furthermore, the *pptrb* mutants exhibited higher transcript levels of genes containing *telo*‐box motifs near the TSS, particularly those encoding ribosomal proteins.

### Loss of PpTRBs results in telomere shortening

To determine whether the function of plant‐specific TRB proteins in telomere maintenance is conserved in mosses, as observed in *attrb1‐3* (Schrumpfová et al., 2014; Zhou et al., 2018) but not in *attrb4‐5* (Amiard et al., 2024), telomere lengths were analyzed in *P. patens* mutants lacking TRB proteins.

Terminal restriction fragment (TRF) analysis was performed on DNA isolated from WT and *pptrb* single‐ and double‐mutant lines across three consecutive passages of protonemas, beginning with the third passage. This design allowed us to investigate whether possible telomere shortening progressed over time, as previously observed, for example, in *attrb1* mutants (Schrumpfová et al., 2014).

**Figure 4:**
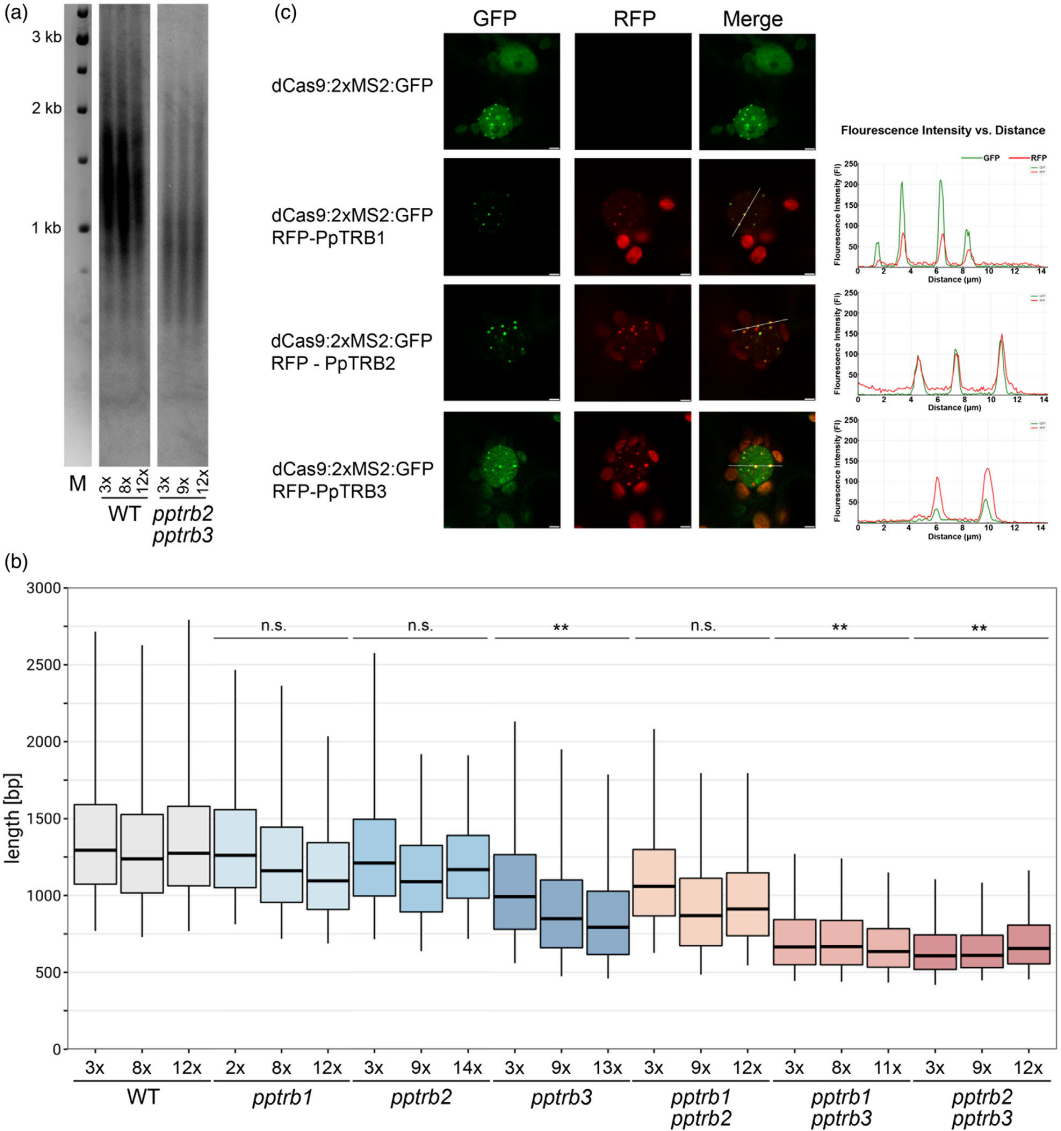
Telomere length analysis reveals stable telomere shortening in some of the *pptrb* mutant lines. (a) Telomere restriction fragment (TRF) Southern blot analysis of three biological replicates of *Physcomitrium patens* wild‐type (WT) and *pptrb2 pptrb3* mutants across the indicated protonemal passages. Molecular weight DNA markers (in kb) are shown. (b) Boxplots represent mean TRF length distributions across three analyzed passages. The graphical representation was generated using WALTER (Lyčka et al., 2021). Shortened telomeres were detected predominantly in the *pptrb1 pptrb3* and *pptrb2 pptrb3* double‐mutant lines, with a median length of approximately 600 bp. Statistics were evaluated using a linear model of medians and the GLHT procedure similar to the Dunnett test (contrast to WT). Benjamini‐Hochberg *P*‐value correction. Asterisks (**) represent *P* < 0.01; n.s. not significant. (c) Simultaneous visualization of telomere‐specific sgRNA (dCas9:2xMS2:GFP) and the RFP‐tagged PpTRBs (pB7WGR2). Intensity profiles corresponding to regions of interest (indicated by white line) confirm overlapping fluorescence signals, indicating co‐localization of PpTRBs with telomeres.

Genomic DNA was digested by the *Mse*I restriction enzyme, and intact telomeric fragments were visualized via Southern hybridization with radioactively labeled telomeric probes (Figure 4a). The median telomere length was quantified using the Web‐based Analyzer of the Length of Telomeres (WALTER) toolset (Lyčka et al., 2021). Telomere length was analyzed not only in the lines analyzed by QuantSeq analysis (WT, *pptrb1 #5, pptrb2 #11, pptrb3 #2, pptrb1 pptrb2 #3, pptrb1 pptrb3 #6, pptrb2 pptrb3 #16*), but also in other single‐ and double‐mutant lines (Figure S10). Telomere lengths are significantly shortened in two double‐mutant lines, *pptrb1 pptrb3* and *pptrb2 pptrb3*, both of which contain mutations in the *PpTRB3* gene (Figure 4b). The median telomere length in these mutant lines was approximately 650 bp, roughly half the length of a WT plant telomere, which exhibited a median length of approximately 1250 bp. Interestingly, this telomere shortening appeared to occur prior to the first analyzed passage and did not progress further across subsequent passages of protonema. In contrast, no significant changes in the telomere lengths were observed in single *pptrb1* and *pptrb2* mutant lines, while significant changes were observed in the *pptrb3* mutant line.

To further investigate PpTRB association with telomeric regions, we performed simultaneous visualization of telomeric tracts and PpTRB proteins. Telomeres were labeled with a telomere‐specific sgRNA construct (dCas9:2xMS2:GFP; Khosravi et al., 2020), while PpTRBs were expressed as RFP fusions (pB7WGR2) under the CaMV 35S promoter (Karimi et al., 2002) in *Nicotiana tabacum* epidermal cells. Intensity profile analyses confirmed overlapping fluorescence signals, demonstrating partial co‐localization of PpTRBs with telomeric foci (Figure 4c).

Altogether, our results demonstrate that PpTRBs localize to telomeric foci within the nucleus and that loss of PpTRB function leads to telomere shortening in several mutant lines. These findings indicate that TRB proteins play a critical role in telomere maintenance in mosses, suggesting a conserved ancestral function present in the last common ancestor of tracheophytes (vascular plants) and bryophytes (non‐vascular plants) that diverged during the Cambrian, 515–494 Ma (Harris et al., 2022).

### PpTRB proteins show conserved nuclear and nucleolar localization

**Figure 5:**
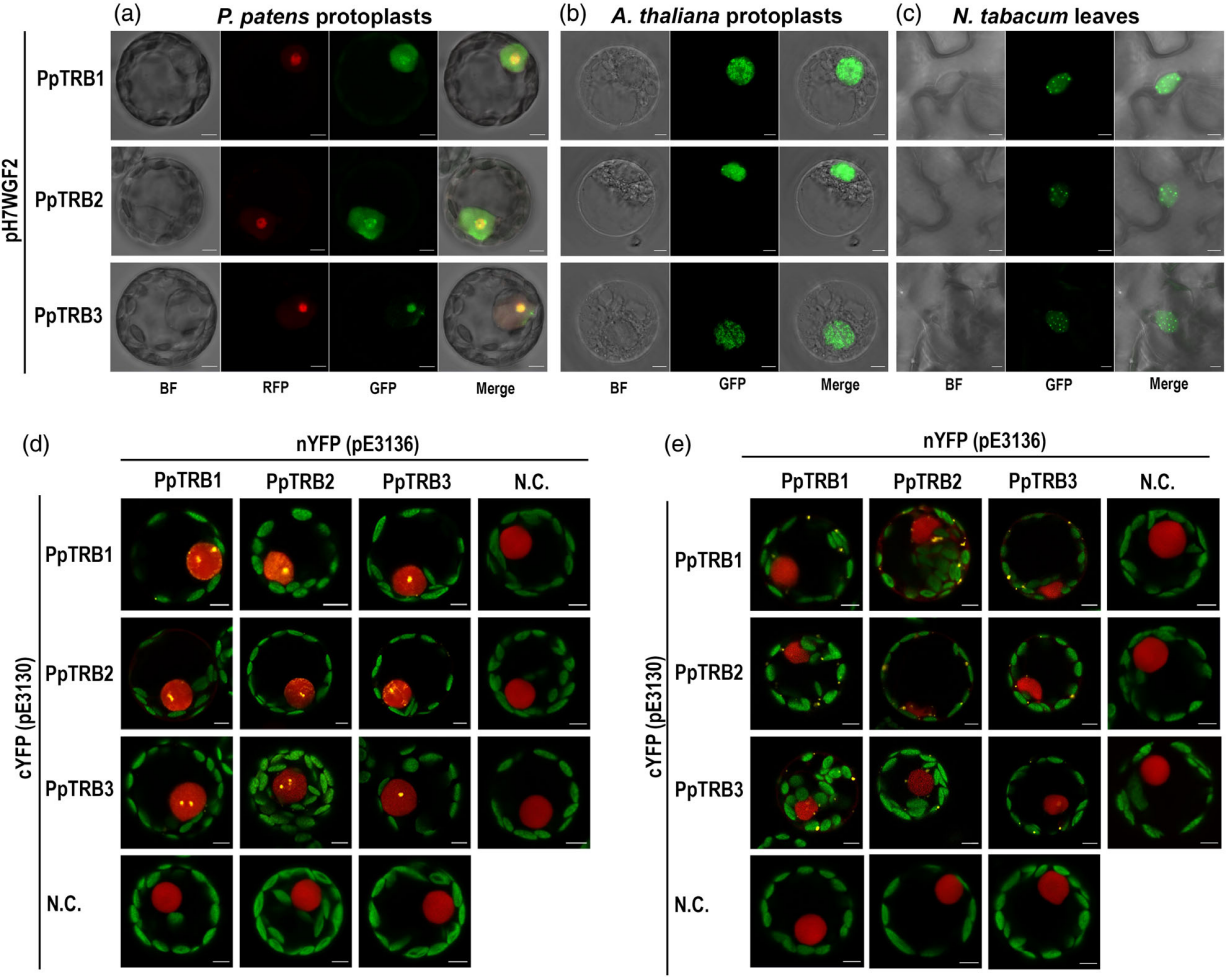
PpTRBs localize to the nucleus across different plant cell types from various organisms and exhibit mutual interactions. (a–c) Subcellular localization of PpTRB1–3 proteins fused to GFP, transiently expressed in: (a) *Physcomitrium patens* protoplasts derived from 7‐day‐old protonema, (b) *Arabidopsis thaliana* protoplasts derived from seedling roots, (c) *Nicotiana tabacum* (SR1) leaf epidermal cells. In all examined cell types, PpTRB proteins localize predominantly to the nucleus, with minor variations observed between species. In *P. patens* protoplasts, PpTRB proteins were co‐expressed with mRFP‐Fibrillarin and merged channels of Bright Field (BF), GFP and RFP are shown. For *A. thaliana* and *N. tabacum*, merged channels of Bright Field and GFP are shown. Scale bars = 5 μm. (d, e) Protein–protein interactions among PpTRBs, fused with nYFP or cYFP fragments, were detected using Bimolecular Fluorescence Complementation (BiFC) in *P. patens* protonemal protoplasts. Reconstituted YFP fluorescence (protein interaction) is shown together with mRFP fluorescence (internal control for transformation and expression) as merged confocal images. As negative controls, PpTRB proteins were co‐expressed with corresponding BiFC vectors expressing solely nYFP or cYFP. (d) Fluorescence foci of varying numbers and sizes are localized primarily in the nucleus, (e) in some protoplasts, fluorescence foci are also observed in the cytoplasm and at the cellular periphery. For individual fluorescence channels corresponding to Figure 5d,e, see Figures S12 and S13, respectively. Scale bars = 5 μm.

To determine the subcellular localization of TRB proteins in mosses, we generated translational fusions of PpTRB1, 2, and 3 proteins with green fluorescent protein (GFP). These constructs were placed under the control of the constitutive CaMV 35S promoter (35S::GFP:PpTRB1‐3) (Karimi et al., 2002). Using polyethylene glycol (PEG)‐mediated transient transformation of *P. patens* protoplasts derived from 7‐day‐old protonema cells, we observed that PpTRBs preferentially localized to the nucleus and nucleolus (Figure 5a). Specifically, PpTRB1 and PpTRB2 proteins are localized within both the nucleus and nucleolus, whereas PpTRB3 was exclusively localized to the nucleolus. This subcellular distribution in *P. patens* was consistent with the localization of AtTRB1‐3 proteins in *Arabidopsis* cells (Dvořáčková et al., 2010) and the localization of AtTRB1‐4, but not AtTRB5, in tobacco leaf epidermal cells (Kusová et al., 2023; Zhou et al., 2016). To confirm nucleolar localization, *P. patens* protoplasts were co‐transfected with the marker construct mRFP‐Fibrillarin1 (Pih et al., 2000), which labels the nucleolus. Clear co‐localization of PpTRB3 with mRFP‐Fibrillarin1 confirmed its nucleolar enrichment (Figure 5a; Figure S11).

To further investigate the subcellular localization, we expressed PpTRB‐GFP constructs in *A. thaliana* and *N. tabacum* cells. PEG‐mediated transient transformation of *A. thaliana* protoplasts, derived from seedling roots, revealed that PpTRBs predominantly localized to the nucleus, though with a more dispersed pattern compared with *P. patens* protoplasts (Figure 5b). In contrast, transient *Agrobacterium*‐mediated transformation of *N. tabacum* (cv. Petit Havana SR1; referred to hereafter as SR1) leaf epidermal cells also showed nuclear localization of PpTRBs, albeit with a slightly different distribution compared with both *P. patens* and *A. thaliana* protoplasts (Figure 5c).

Overall, the PpTRBs demonstrate preferential nuclear localization being conserved in *Physcomitrium*, *Arabidopsis*, and *Nicotiana* with only subtle species‐specific variations. Notably, the localization pattern of PpTRBs in *P. patens* protoplasts resembles that of AtTRB1‐3 in *Arabidopsis* cells (Dvořáčková et al., 2010).

### PpTRBs mutually interact in the nucleus and/or nucleolus, as well as at cytoplasm and cellular periphery

To investigate whether the mutual interactions observed among all five AtTRB proteins (Kusová et al., 2023; Mozgová et al., 2008) are conserved also in moss, we employed a bimolecular fluorescence complementation (BiFC) assay. The coding sequences of *PpTRB1‐3* genes were fused to sequences coding for N‐ and/or C‐terminal fragments of yellow fluorescent protein (nYFP and cYFP). Protoplasts derived from 7‐day‐old protonema cells were co‐transformed with the mRFP−VirD2NLS construct, which marks the nucleus (Citovsky et al., 2006). Our observations revealed that all three PpTRB proteins form homo‐ and hetero‐dimers, as evidenced by nucleoplasmic fluorescence foci of varying numbers and sizes localized predominantly in the nucleus (Figure 5d; Figure S12). Interestingly, fluorescence foci were also observed in approximately one‐third of the analyzed protoplasts, as well as in the cytoplasm and at the cellular periphery (Figure 5e; Figures S13 and S14).

**Figure 6:**
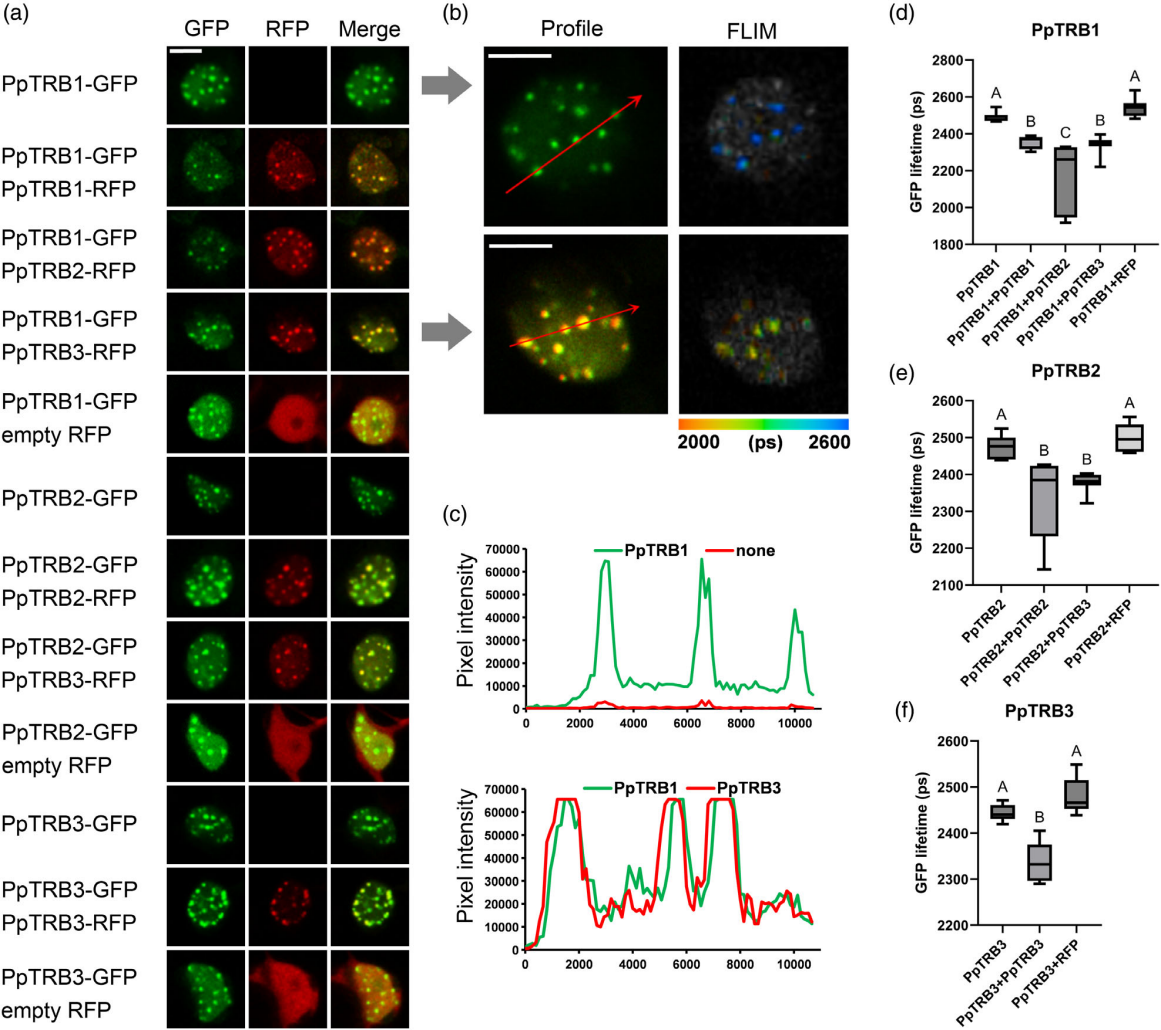
PpTRBs nuclear speckles locate and interact at the same spots in nucleoplasm. (a) Representative confocal images of subnuclear localization of PpTRBs‐GFP/RFP with merged images. (b) PpTRB1‐GFP and PpTRB3‐RFP form nuclear speckles and (c) co‐localize at the same foci; scale bars = 5 μm. (c) Intensity plots for regions of interest (red arrows) indicate co‐localization of PpTRB1 with PpTRB3 in nuclear speckles. For intensity plots of individual samples see Figure S15. (d–f) GFP fluorescence lifetime measured in a FLIM‐FRET interaction assay using the indicated vector combinations transiently expressed in *Nicotiana tabacum* leaves. Error bars represent means ± SD; *n* = 10, and letters indicate statistical significance (two‐way anova and Tukey's *post hoc* test).

To further validate the mutual interactions of PpTRBs in plant cells, PpTRBs were fused with either green or red fluorescent proteins (GFP, RFP). Due to the challenge of measuring fluorescence lifetime in partially swirling protoplasts in solution, these analyses were performed on transiently transformed *N. tabacum* SR1 leaf epidermal cells. Co‐localization of GFP and RFP signals implied possible interaction of PpTRB1–3 proteins in nucleoplasmic foci of varying numbers and sizes (Figure 6a–c; Figure S15). The specific protein–protein interactions at subcellular precision were confirmed using Fluorescence Lifetime Microscopy—Förster Resonance Energy Transfer (FLIM‐FRET; Figure 6d–f).

We further analyzed the GFP lifetime in the nucleosol and compared it with signals from speckles. As shown in the Figure S16, the interaction in speckles appears to be more pronounced than in the nucleosol, although the differences are not substantial. Pairwise comparisons revealed no statistically significant difference between these two compartments; however, significant differences emerged when comparing with the negative control, with the speckles showing a markedly stronger effect.

Overall, our analyses demonstrate that PpTRB proteins in *P. patens* exhibit mutual interactions similar to those observed in AtTRB proteins. The interactions of PpTRBs are predominantly nuclear and localized in nucleoplasmic foci, resembling the behavior of AtTRB1–4. However, similar to AtTRB5, whose homodimeric interactions are predominantly cytoplasmic (Kusová et al., 2023), PpTRBs also exhibit interactions in the cytoplasm, including a moderate enrichment near the cellular periphery, in a subset of *P. patens* protoplasts.

### Phylogeny of plant TRB proteins

Although we previously identified a single plant‐specific member of the TRB family in *Klebsormidium nitens* (Klebsormidiophyceae) (Kusová et al., 2023), TRB presence in other classes of streptophyte algae (Mesostigmatophyceae, Chlorokybophyceae, Charophyceae, Charophycae, Coleochaetophyceae), hornworts or liverworts within Bryophyta, remained unresolved. To trace the evolutionary history of TRB proteins in Streptophyta, we surveyed for TRB homologs across fully sequenced and annotated genomes, a proteome, as well as newly available transcriptomes, representing key groups within Streptophyta.

We probed 11 annotated genomes, proteomes, and 21 previously assembled transcriptomes. We further assembled 30 transcriptomes from publicly available data. Genomes and transcriptomes were evaluated for BUSCO sequence completeness. Our dataset consisted of 444 sequences (see Table S3). In several instances, one or several of the three domains defining TRBs (H1/5‐like, MYB, coiled‐coil) were missing. The coiled‐coil motif seems to be the most diverse at the sequence level, showing large variations and clade‐specific features. When probing reference proteomes, we identified, in several instances, a full‐length TRB associated with a truncated copy within the same species (e.g., *Nitella flexilis*), which could indicate pseudogenization. However, the presence of truncated versions could in some cases be ascribed to genome annotation errors as we were able to obtain transcripts of the canonical architecture in closely related species for these duplicated genes.

**Figure 7:**
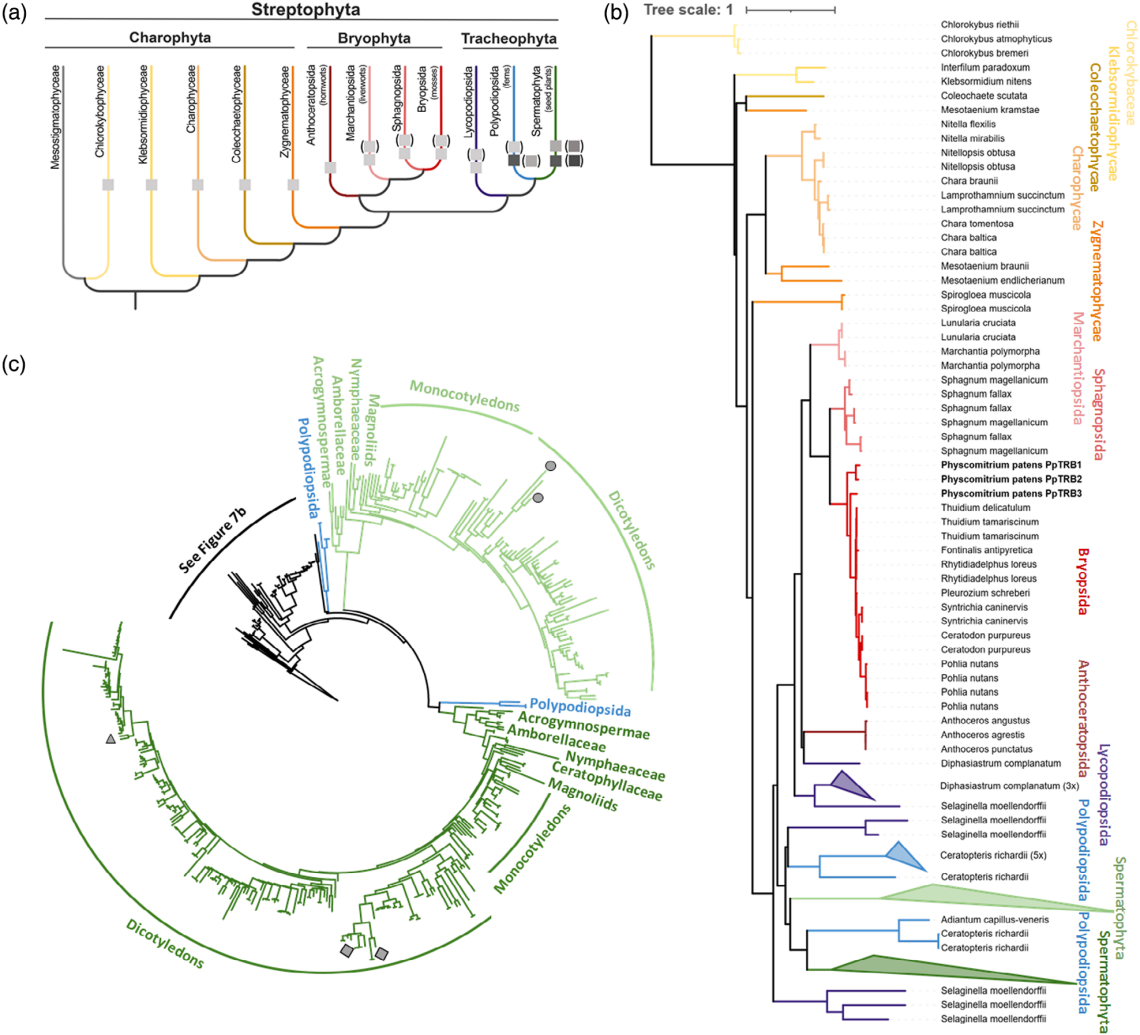
Phylogenetic analysis of Telomere repeat binding (TRB) proteins across Streptophyta. (a) A simplified evolutionary overview illustrating the major lineages within Streptophyta containing identified TRB proteins. The emergence of a single TRB protein is first observed in streptophyte algae, specifically within the Chlorokybophyceae. Subsequent whole‐genome duplication events contributed to the diversification of TRB proteins into several homologous variants in mosses and lycophytes. In Spermatophyta (seed plants), further diversification resulted in the presence of multiple distinct TRB homologs, which cluster into two major evolutionary lineages. The evolutionary framework is adapted from (Rensing, 2020) and (Cheng et al., 2019). (b) Distribution of TRB proteins across various streptophyte taxa, with a particular focus on streptophyte algae and bryophytes. In bryophyte lineages, such as Marchantiopsida, Bryopsida, and Sphagnopsida, TRB proteins form a monophyletic group. In contrast, TRB proteins in Spermatophyta exhibit greater diversification. (c) In Spermatophyta, TRB proteins have diverged into two distinct clades, indicated by light and dark green branches. The *Arabidopsis thaliana* paralogs AtTRB1 (triangle), AtTRB2, and AtTRB3 (squares) are grouped within the dark green branch, while AtTRB4 and AtTRB5 (circles) belong to the light green branch (Kusová et al., 2023).

Within the most basal lineages of Streptophytes, TRBs were observed in Chlorokybophyceae, but we failed to detect them in the sister clade of Mesostigmatophyceae (Figure 7a). We successfully retrieved TRBs from four out of five cryptic species of *Chlorokybus*. Here, the TRB structure was unusual, showing a disordered C‐terminal coiled‐coil motif.

The TRB phylogeny demonstrated a strong departure from the established streptophyte topology especially in charophytes. Although, both Klebsormidiophyceae and Charophyceae were resolved as monophyletic (Figure 7b; Figure S17), we recovered Zygnematophyceae as polyphyletic. Within Zygnematophytes, we failed to get a positive match in several representatives within the Desmidiales order. It is noteworthy that Desmidiales were characterized by a large proportion of missing BUSCO genes (Table S3) which might indicate a severe genome contraction leading to the loss of *TRB*. Moreover, *Spirogloea muscicola* branched in proximity to embryophytes, fitting the recent hypothesis of a close relationship between these two groups and the species divergence from other members of the Zygnematophyceae (Cheng et al., 2019; Hess et al., 2022).

Within bryophytes, Marchantiopsida (liverworts), Bryopsida (joint‐toothed mosses), Sphagnopsida (peat mosses), each formed a monophyletic group with several independent duplications located within them. Anthocerotopsida (hornworts) was positioned as bryophytes' sister clade as expected (One Thousand Plant Transcriptomes Initiative 2019; Leebens‐Mack et al., 2019). The evolutionary patterns were more complex within Lycopodiopsida (lycophytes) where a total of six putative TRBs from *Selaginella moellendorffii* branched at three distinct positions: at the base of embryophytes, sister to bryophytes and sister to Polypodiopsida (ferns). However, the other representative of lycophytes in our dataset, *Diphasiastrum complanatum*, possessed only TRBs that were associated with bryophyte TRBs. The unexpected positions of *S. moellendorffii* paralogs could be explained by common phylogenetic reconstruction artifacts (e.g., long‐branch attraction, model misspecification) or they could hint at a more complex evolutionary history of *TRB*s including horizontal gene transfer or gene loss in other clades.

The placement of *PpTRB3* as sister to the other Bryopsid sequences, renders the *PpTRB* set paraphyletic. This unexpected topology can be explained by differential gene retention and additional duplications following the ψ WGD event during the early history of Bryopsida (Gao et al., 2022). One copy has survived in all Bryopsids (*PpTRB3*), whereas the other was duplicated in the *P. patens* lineage and lost in the common ancestor of the other sampled Bryopsids.

Our phylogeny further demonstrated that a *TRB* duplication occurred before the divergence of Spermatophyta, and in particular, before the divergence of Angiosperms (Figure 7c; Figure S17). The established sister clade relationship between Acrogymnospermae and Angiospermae was recovered. The topology of the Acrogymnospermae subtree suggested a gene duplication within the Pinopsida family. The relationships within Monocots and Eudicots were generally consistent with the accepted phylogeny. We confirmed the Eudicots' gene duplication within the lineage containing the *AtTRB1‐3* paralogs. Despite sharing all its nested WGDs with closely related species (e.g., *Arabidopsis lyrata* and *Arabidopsis arenosa*), *A. thaliana* was the only species in our dataset containing the full complement of TRB copies.

In summary, our analysis indicates that TRB proteins originated in Chlorokybophyceae and are present across streptophyte algae. In bryophytes, TRBs form a monophyletic group, whereas in seed plants they diversified into two distinct groups following an early duplication event.

## DISCUSSION

In contrast to animals and yeast, plants have evolved a unique family of proteins known as TRBs, characterized by a distinct domain architecture comprising an N‐terminal Myb‐like domain, an H1/5‐like domain, and a C‐terminal coiled‐coil domain. In seed plants, TRB proteins play a pivotal role, particularly in the structure and maintenance of telomeres, and regulation of gene expression via epigenetic complexes associated with gene regulatory elements (Kusová et al., 2023; Schrumpfová et al., 2004, 2016; Zhou et al., 2016).

To date, the function of TRB proteins has been extensively characterized only in seed plants, particularly in *A. thaliana* (Amiard et al., 2024; Dreissig et al., 2017; Kusová et al., 2023; Schrumpfová et al., 2004, 2008, 2014, 2016; Teano et al., 2023; Wang et al., 2023; Zhou et al., 2018) and partially in apple (*Malus domestica*) (An et al., 2021) and rice (Xuan et al., 2024). To fill in missing knowledge on these proteins in earlier branching land plants, we characterized here TRB proteins by examining their function in *P. patens*.

### Impact of *pptrb* mutations on moss development

One of the key evolutionary innovations in land plants was the shift from two‐dimensional (2D) to three‐dimensional (3D) growth (Moody, 2022). This 3D growth is a hallmark feature of land plants. In the moss *P. patens*, a representative of the earliest lineage of land plants, 3D growth (gametophore) is preceded by an extended 2D filamentous growth (protonema) phase, although the former is not a prerequisite for survival in *P. patens* (Moody, 2019).

In this study, we demonstrate that in the moss *P. patens*, three highly similar TRB proteins are transcribed during the 2D protonemal stage. In contrast to mosses, seed plants predominantly exhibit two main diverging lineages of TRBs that occurred before the divergence of Spermatophyta, and in particular, before the divergence of Angiosperms. In the seed plant *A. thaliana*, five paralogs cluster into two lineages (AtTRB1–3 and AtTRB4–5), with growth or developmental phenotypes emerging only when an entire lineage is disrupted (Amiard et al., 2024; Kusová et al., 2023; Schrumpfová et al., 2014; Wang et al., 2023; Zhou et al., 2018).

Our findings suggest that in *P. patens*, TRB proteins are essential for cell viability, as we were unable to generate a plant mutant for all three PpTRB proteins. Even single knockouts for *PpTRB2* and *PpTRB3* show significant phenotypic changes, including reduced 3D stage and gametophore formation. We were unable to achieve a complete knockout of *PpTRB1* using HR, as only knockdown lines generated via CRISPR‐Cas were obtained. In *pptrb3* mutants and all double‐mutant lines, the apical caulonemal cells, which play a crucial role in the transition to differentiated cells forming gametophores, were frequently defective, presenting premature senescence and progressing to cell death.

Whether the essentiality of TRB proteins is linked to their role in early moss development, particularly in regulating the transition from 2D protonemal stages to 3D gametophore formation, and in maintaining apical caulonemal cell viability, remains to be further investigated.

### Functional conservation and divergence of PpTRBs

TRB proteins were first identified in maize and *A. thaliana* for their ability to bind telomeric DNA (Marian et al., 2003; Schrumpfová et al., 2004). This role was confirmed by telomere shortening in *Arabidopsis* mutants lacking *AtTRB1–3*, but not *AtTRB4–5* (Amiard et al., 2024; Lee & Cho, 2016; Schrumpfová et al., 2014).

Our findings indicate that TRB proteins are involved in telomere maintenance not only in flowering plants but also in mosses. Telomere shortening was observed in *pptrb* mutant lines, specifically those with mutations in the *PpTRB3* gene (*pptrb3*, *pptrb1 pptrb3*, and *pptrb2 pptrb3*). These findings are consistent with findings in *Arabidopsis* plants mutants lacking AtTRB1 (Schrumpfová et al., 2014) and AtTRB1‐3 (Zhou et al., 2018). The significance of these findings lies in the fact that TRB proteins in *P. patens* differ from the two distinct groups of TRBs characterized in seed plants. Telomere shortening, indicative of chromosome instability (Goffová et al., 2019), was also associated with morphological changes in the *P. patens* mutant lines. The absence of telomere shortening in single mutants suggests possible functional redundancy among the three highly similar TRB proteins in *P. patens*.

In addition to binding telomeric DNA, *A. thaliana* TRBs also associate with promoter regions of various genes containing *telo*‐boxes, Site II motifs, or JMJ14 binding motifs, likely contributing to epigenetic regulation (Amiard et al., 2024; An et al., 2021; Kusová et al., 2023; Schořová et al., 2019; Schrumpfová et al., 2016; Tan et al., 2018; Wang et al., 2023; Xuan et al., 2024; Zhou et al., 2018). To elucidate the molecular mechanisms underlying the observed phenotypic changes, we conducted a differential expression analysis using RNA‐seq data. The identification of DEGs in these mutants provided insights into the cellular pathways affected by TRB protein loss. Notably, several genes related to regulation of DNA‐templated transcription or various stress responses were significantly altered, highlighting the diverse roles TRB proteins play in maintaining cellular homeostasis. Interestingly, the *pptrb* mutants exhibited higher transcription levels of genes containing *telo*‐box motifs near TSS, particularly those encoding ribosomal proteins. However, genes with highly variable baseline expression levels, ranging from very low to very high as identified by DESeq, did not exhibit consistent changes. In other analyzed motifs already described as to be recognized by TRB proteins as Site II (TGGGCY) (Hervé et al., 2009), and JMJ14 binding motifs (CTTGnnnnnCAAG) (Zhang et al., 2015), no difference was observed between the motifs in the promoters of the genes showing high transcription in WT and in mutants. Additionally, none of the motifs associated with plant hormones, such as cytokinin (ARR10 (GATY)) (Hosoda et al., 2002), ethylene (GCC‐box (GCCGCC)) (Hao et al., 1998), and ABA (ABRE (ACGTG)) (Hattori et al., 2002) showed higher occurrence in the promoters of the highly transcribed genes in the *pptrb* mutants.

### Nuclear localization and interaction of TRBs

These findings suggest that PpTRBs in various plant cells (*P. patens*, *A. thaliana*, *N. tabacum* or *N. benthamiana*) predominantly localize to the nucleus. The nuclear localization of PpTRBs in *A. thaliana* protoplasts closely resembles the localization of native AtTRBs in isolated *Arabidopsis* nuclei, as observed using anti‐TRB antibodies (Kusová et al., 2023). Mutual interactions of PpTRBs were predominantly detected in the nucleus, where they were concentrated within nucleoplasmic foci. This pattern is consistent with the behavior of most AtTRBs in *A. thaliana* and *N. benthamiana* cells (Dvořáčková et al., 2010; Kusová et al., 2023).

The mutual PpTRB interactions were observed using BiFC, a technique that may also detect indirect interactions, as previously demonstrated for TERT and Recombination UV B‐like (RUVBL) proteins, plant homologs of Pontin and Reptin, whose interaction is mediated by TRB proteins (Schořová et al., 2019). Additionally, mutual interactions were also confirmed using FLIM‐FRET. Since energy transfer between fluorophore groups can only occur efficiently when the interacting proteins are in close physical proximity (≤10 nm), it is highly likely that PpTRBs directly interact. This nuclear localization and interaction pattern supports the hypothesis that TRB proteins have a conserved role in the nucleus, potentially contributing to telomere maintenance and other nuclear processes.

Interestingly, in a subset of *P. patens* protoplasts, mutual PpTRB interactions were also observed in the cytoplasm, including the cellular periphery. This localization pattern resembles that of AtTRB5 in *N. benthamiana*, which is predominantly cytoplasmic (Kusová et al., 2023). TRB proteins are multifunctional and participate in diverse protein complexes (Amiard et al., 2024; An et al., 2021; Kusová et al., 2023; Schořová et al., 2019; Schrumpfová et al., 2008, 2014; Tan et al., 2018; Wang et al., 2023; Xuan et al., 2024; Zhou et al., 2016, 2018). For example, TRBs directly interact with the catalytic subunit of telomerase (TERT) and POT proteins, and indirectly with the dyskerin homolog AtCBF5. Notably, some of these proteins also exhibit mutual interactions in cytoplasmic foci (Schořová et al., 2019).

Furthermore, GO analysis of moss mutants lacking TRB proteins revealed an enrichment of terms such as ‘cell periphery’ and ‘plasma membrane’. Although this cytoplasmic localization may partly result from the overexpression of the proteins used in these experiments, the relationship between these phenomena remains unclear and warrants further investigation.

### Evolutionary insights into TRB proteins

A comprehensive phylogenetic analysis encompassing not only mosses, but also streptophyte algae, hornworts, liverworts, and seed plants demonstrated that genes encoding TRB proteins originated in early‐diverging Streptophyta. Transcriptomics data from species lacking assembled genomes were utilized to confirm the absence of detectable TRB gene homologs in the transcriptomes of early‐diverging lineages.

In this study, we discovered that Chlorokybaceae, but not Klebsormidiophyceae as we previously mentioned (Kusová et al., 2023), is the earliest diverging lineage within Streptophyta in which plant TRB genes could be detected. However, in certain basal representatives, doubts remain regarding the presence of full‐length transcripts containing all three defining domains or their correct structure, due to sequence divergence and variability in transcriptome data completeness or quality. Additionally, the reliability of transcriptomic data is influenced by the potential for contamination, which may arise during RNA extraction, sequencing, or deconvolution. To mitigate these issues, we compared transcriptomes from closely related species generated from different Bioprojects, enabling the identification of contamination where possible.

The presence of TRB genes was subsequently newly identified in other streptophyte algal lineages, including Charophyceae, Coleochaetophyceae, and Zygnematophyceae. Among mosses (Bryophyta), the number of TRB genes varied and appeared to be associated with multiple independent ancient WGD events (Gao et al., 2022). These events likely occurred across several classes, including Marchantiopsida (liverworts), Sphagnopsida (peat mosses), Bryopsida (joint‐toothed mosses), and Anthocerotopsida (hornworts) (Clark & Donoghue, 2018; Gao et al., 2022).

In contrast to mosses, seed plants predominantly possess two major diverging lineages of TRBs (Kusová et al., 2023). These lineages likely arose from WGD events that expanded the TRB family to as many as 10 members, with increased copy numbers observed in polyploid species such as triploids and tetraploids. Unusual branching patterns in seed plants can be explained by the usual phenomena of model inadequacy and pernicious incomplete lineage sorting. Furthermore, these observations could be influenced by errors inherent in automated genome annotation, which remain prone to gene delineation inaccuracies even when trained on transcriptome data (Salzberg, 2019).

Our findings suggest that the evolutionary origins of TRB homologs may predate current estimations. However, a more comprehensive analysis of Chlorophyta and other eukaryotic lineages, such as Excavata, is required. Additionally, genetic manipulation will be necessary to fully elucidate the extent of functional conservation of TRBs across Streptophyta.

## MATERIAL AND METHODS

### Plant material cultivation and morphological analysis

The WT ‘Gransden’ strain of *P. patens* (Hedw.) B.S.G. (Rensing et al., 2008) was used for the generation of all *pptrb* mutants. The moss lines were cultured as ‘spot inocula’ on BCD agar medium supplemented with 1 mM CaCl_2_ and 5 mM ammonium tartrate (BCDAT medium), or as lawns of protonema filaments by subculturing homogenized tissue on BCDAT agar overlaid with cellophane in growth chambers with an 18/6 h day/night cycle at 22°C/18°C (Cove et al., 2009). Homogenized or unhomogenized protonema were grown for 1 week in normal light conditions on plates where 0.5% sucrose was included in the medium with 1.5% agar and then transferred to the dark for 3 weeks. Petri dishes were positioned vertically to enhance the observation of caulonema growth.

After 1 month of growth on BCD or BCDAT, whole colonies of WT and *pptrb* mutants, were photographed using a Canon EOS77D camera equipped with a Canon EF 28–135 mm f/3.5–5.6 objective. Gametophore details were captured using a Leica M205FA stereomicroscope with a Plan‐Apochromat 2.0× objective. Staining with 10 μg ml^−1^ PI (Sigma‐Aldrich, St. Louis, MO, USA) was performed as described previously (Lelkes et al., 2023). Images of caulonema growth in the dark were obtained by an Epson PERFECTION V700 PHOTO scanner and by a Zeiss AxioZoom.V16‐Apotome2 stereo zoom microscope with a Plan‐Neofluar 1× or a Plan Apo 0.5× objective.

### Construction of *pptrb* mutant lines

The STOP codon knock‐in was introduced by homology‐directed repair following Cas9 induction of a double‐strand break within the *PpTRB1* locus. A 50 bp double‐stranded DNA donor template containing the desired mutation was used for repair. The donor template was composed of two complementary oligonucleotides (pKA1364 and pKA1365; Table S4) homologous to the first exon of the *PpTRB1* locus. The oligonucleotides introduced a substitution of +63G to C (creating a *SalI*I cleavage site), deletion of +69TG, and a substitution of +71G to A, collectively generating a STOP codon at the position of the 23rd amino acid (Figure S1a). Gateway destination vector pMK‐Cas9‐gate (Addgene plasmid #113743), which includes a Cas9 expression cassette and kanamycin resistance, along with the entry vector pENTR‐PpU6sgRNA‐L1L2 (Addgene plasmid #113735) containing the PpU6 promoter, and sgRNA scaffold, was sourced from (Mallett et al., 2019). PpTRB1‐specific sgRNA spacer was synthesized as two complementary oligonucleotides (pKA1174 and pKA1275; Table S4). Four nucleotides were added to the 5′ ends of the oligonucleotides to create sticky ends compatible with *Bsa*I‐linearized pENTR‐PpU6sgRNA‐L1L2 upon annealing. The Cas9/sgRNA expression vector was assembled using the Gateway LR reaction to recombine the entry vector pENTR‐PpU6sgRNA‐L1L2 with the TRB1 sgRNA spacer and destination vector pMK‐Cas9‐gate. This DNA construct was co‐transformed with the donor template (annealed pKA1364+pKA1365) into protoplasts by PEG‐mediated transformation (Liu & Vidali, 2011). The sgRNA was designed in the CRISPOR online software, using *P. patens* (Phytozome V11) as the genome and *Streptococcus pyogenes* (5′ NGG 3′) as the PAM parameter. The protospacer with the highest specificity score was selected for further experiments. After 5 days of regeneration, the transformed protoplasts were transferred to the BCDAT medium supplemented with 50 mg L^−1^ G418. Following 1 week of selection, the G418‐resistant lines were propagated. Crude extracts from young tissues of these lines were used for PCR amplification of genomic DNA around editing sites using primers pKA1383, and pKA1384. The PCR products were digested by *Sal*I and analyzed by DNA electrophoresis. Lines with cleaved PCR products were subsequently analyzed to confirm the accurate introduction of mutations. Four lines, *pptrb1 #5, #6, #10* and *#12*, were identified with correctly integrated STOP codons. All the lines exhibited very similar growth and morphology phenotypes.

Constructs for gene targeting of *PpTRB2* and *PpTRB3* were assembled in GoldenBraid cloning system (Sarrion‐Perdigones et al., 2011). Each construct included a selection cassette (NPTII or Hyg^R^) flanked by approximately 1 kb of sequences from each of the 5′‐ and 3′ regions of the target genes (Figure S1b,c). A total of 30 μg of linear DNA from each construct was introduced into protoplasts using PEG‐mediated transformation (Liu & Vidali, 2011). After 5 days of regeneration, the transformed protoplasts were transferred to Petri dishes containing the BCDAT medium supplemented with 30 mg L^−1^ hygromycin or 50 mg L^−1^ G418. Following three rounds of selection, the transformants were deemed stable. Gene replacement was confirmed by PCR of genomic DNA isolated from the stable transformants, following the method described by Dellaporta et al. (1983). Primers were designed to amplify the selection cassette in an outward direction, pairing with gene‐specific primers that annealed to sequences outside the homology regions integrated into the targeting vectors (Figure S1b,c; Table S4). A single representative line for each confirmed mutant, *pptrb1 #5*, *pptrb2 #11 and pptrb3 #2*, was selected for further investigation.

Double mutants were prepared and verified as described above, as follows: (i) *pptrb1 pptrb2* mutants were prepared by delivering *PpTRB1* CRISPR/Cas9 construct into protoplasts isolated from *pptrb2 #11*; (ii) *pptrb1 pptrb3* mutants were prepared by delivering *PpTRB1* CRISPR/Cas9 construct into protoplasts isolated from *pptrb3 #2*; (iii) *pptrb2 pptrb3* mutants were prepared by delivering *PpTRB3* replacement vector into protoplasts isolated from *pptrb2 #11*. From two to three independent double‐mutant lines were investigated, and a single representative line was subsequently selected for further detailed investigation: *pptrb1 pptrb2 #3*, *pptrb1 pptrb3 #6*, and *pptrb2 pptrb3 #16*.

### RNA isolation and QuantSeq 3′mRNA‐seq

The total RNA was isolated from 7‐day‐old protonemal tissue (WT, *pptrb1 #5, pptrb2 #11, pptrb3 #2, pptrb1 pptrb2 #3, pptrb1 pptrb3 #6, pptrb2 pptrb3 #16*). Approximately, 100 mg of tissue per sample was homogenized using glass beads. About 1 ml of TRI reagent (TRI REAGENT^®^; MRC) was added and incubated for 5 min at room temperature. Then, 200 μl of chloroform was added and mixed, followed by centrifugation for 15 min at 12 000 ***g*** at 4°C. After centrifugation, the upper aqueous phase (appr. 500 μl) was used for RNA purification with Direct‐zol RNA Miniprep Kits (Zymo Research, Irvine, CA, USA), including DNaseI treatment. The integrity of RNA was checked by agarose gel electrophoresis, and concentration was measured using a NanoDrop spectrophotometer (NanoDrop Technologies, Wilmington, DE, USA).

RNA‐seq analysis was carried out by the Core Facility Genomics of CEITEC Masaryk University. RNA integrity was checked on the Fragment Analyzer using the RNA Kit 15 nt (Agilent Technologies, Santa Clara, CA, USA). A total of 400 ng of total RNA was used as an input for library preparation using the QuantSeq 3′mRNA‐Seq Library Prep Kit (FWD) (Lexogen, Vienna, Austria) with polyA selection in combination with the UMI Second Strand Synthesis Module for QuantSeq FWD and the Lexogen i5 6 nt Unique Dual Indexing Add‐on Kit (Lexogen). Library quantity and size distribution were checked using the QuantiFluor dsDNA System (Promega, Madison, WI, USA) and the High Sensitivity NGS Fragment Analysis Kit (Agilent Technologies). The final library pool was sequenced on the NextSeq 500 (Illumina, San Diego, CA, USA) using the High Output Kit v2.5 75 Cycles (Illumina), resulting in an average of 12 million reads per sample.

### Data analysis and GO

The Binary Base Call (BCL) files obtained from QuantSeq 3′ mRNA‐seq were converted to FASTQ format using *bcl2fastq* v. 2.20.0.422 Illumina software for base calling. Quality check of raw single‐end FASTQ reads was carried out by FastQC (1). The adapters and quality trimming of raw FASTQ reads were performed using Cutadapt v4.3 (Martin, 2011) with Illumina adapter trimming and parameters ‐m 35 ‐q 0,20. Trimmed RNA‐seq reads were mapped against the genome of *P. patens* (Ppatens_318_v3.3 and Ppatens_870_v6.1) using STAR v2.7.11 (Dobin et al., 2013) as a splice‐aware short‐read aligner and default parameters except ‐‐outFilterMismatchNoverLmax 0.4 and ‐‐twopassMode Basic.

The differential gene expression analysis was calculated based on the gene counts produced using featureCounts from Subread package v2.0 (Liao et al., 2014) and further analyzed by Bioconductor package DESeq2 v1.34.0 (Love et al., 2014), based on the reference annotation from Ppatens_318_v3.3 and Ppatens_870_v6.1. Data generated by DESeq2 with independent filtering were selected for the differential gene expression analysis due to its conservative features and to avoid potential false‐positive results, PCA was computed from gene expression normalized using DESeq2 Variance Stabilizing Transformation. Genes were considered as differentially expressed (DEG) based on a cutoff of adjusted *P*‐value <0.05 and log2 (fold‐change) ≥1 or ≤−1. Transcript per million (TPM) values were computed using DGEobj.utils convertCounts function. Clustered heatmaps were generated from selected top differentially regulated genes using R package pheatmap v1.0.12, volcano plots were produced using ggplot2 v3.3.5 package and MA plots were generated using ggpubr v0.4.0 package.

GO enrichment analysis was done by g:Profiler version e109_eg56_p17_1d3191d (Raudvere et al., 2019, https://biit.cs.ut.ee/gprofiler) with the default setting using DEGs based on the reference annotation from Ppatens_318_v3.3, as input, and the number of terms used for plotting was reduced based on the results from REVIGO (Supek et al., 2011; http://revigo.irb.hr/) with the predefined cutoff value *C* = 0.5.

The distribution and frequency of promoter motifs in genes with the highest transcript abundance (20th percentile) in WT and *pptrb* mutant plants were analyzed with the GOLEM program (Gene regulatOry eLEMents; https://golem.ncbr.muni.cz) (Nevosád et al., 2025), based on the Ppatens_318_v3.3 reference annotation. GOLEM enables precise localization of motifs relative to the TSS or ATG codon across plant genomes. The 20th percentile was defined following Nevosád et al. (2025), that is, genes whose transcripts collectively account for 20% of the total mRNA abundance from protein‐coding genes at a given developmental stage. TPM values were calculated from 3′ mRNA‐seq data as described in Nevosád et al. (2025) and processed according to the published pipeline, available at https://github.com/sb‐ncbr/golem. The resulting motif distribution data were visualized using a private version of GOLEM (source codes for UI are at GitHub at https://github.com/sb‐ncbr/golem). Additionally, genes identified as DEGs by DESeq2 (based on Ppatens_318_v3.3 annotation) were analyzed for promoter motif distribution and frequency. In this case, TPM normalization was not applied, and all DEGs were included in the analysis irrespective of their transcript abundance.

GOLEM‐related statistics is calculated in three approaches. First, the peaks in the distribution plots are detected using peakfinder (package prcma; Borchers, 2023) and their significance is estimated using a *z*‐score calculating deviation from the mean accounting for residuals modeled by ARIMA (package forecast; Hyndman et al., 2025). Then, Fisher's exact test is calculated to test if the peak value (i.e., number of motifs in such relative position to TSS) relative to the number of genes in the selected percentile is significantly different from the whole genome. If multiple Fisher's exact tests are calculated for multiple lines, the *P*‐values are corrected by the Bonferroni method. The comparisons of mutants with WT are calculated using a generalized linear mixed model (package glmmTMB; Brooks et al., 2025) with a negative binomial distribution, genotype, and bucket distance from TSS as fixed factors and RNA‐seq replicas as a random intercept. The *P*‐values of the comparisons were estimated using emmeans (Lenth et al., 2025) and corrected by the Benjamini‐Hochberg Procedure.

### Cloning of constructs encoding PpTRB proteins

To obtain amplicons with *PpTRB1, PpTRB2, and PpTRB3* genes, total RNA was isolated from 7‐day‐old protonemal tissue using TRI reagent (TRI REAGENT^®^; MRC). cDNA was synthesized from 1 μg of RNA via reverse transcription using SMARTScribe™ Reverse Transcriptase (TAKARA, Shiga, Japan) and oligo dT‐VN primer (Clontech, Mountain View, CA, USA) (Table S4). Expression vectors were constructed using the Gateway^®^ system (Invitrogen, Carlsbad, CA, USA). The constructs encoding PpTRB proteins were amplified from cDNA using KAPA Taq DNA Polymerase (KAPA Biosystems, Wilmington, MA, USA) and specific primers F‐PpTRB1 and R‐PpTRB2(stop) for PpTRB1, F‐PpTRB2 and R‐PpTRB2(stop) for PpTRB2, and F‐PpTRB3 and R‐PpTRB3(stop) for PpTRB3 (Table S4). PCR products were adapted for Gateway cloning in adaptor PCR using attB1/2 primers (Invitrogen) and KAPA Taq DNA Polymerase, and introduced directly to entry Gateway vectors (pDONRZeo, pDONR207) using the BP Clonase™ II enzyme mix (Thermo Fisher Scientific, Waltham, MA, USA). Alternatively, PCR products were introduced into pCR™II‐TOPO^®^ vector (TOPO^®^ TA Cloning^®^ Kit; Invitrogen) and sequenced. TOPO plasmids then served as templates in adaptor PCR or their inserts were recombined to entry vectors using the BP Clonase™ II enzyme mix (Thermo Fisher Scientific) according to manufacturer's instruction.

The resulting amplicons with *PpTRB1‐3* genes were cloned into the pDONR207 entry vector (Thermo Fisher Scientific) using the BP Clonase™ II enzyme mix (Thermo Fisher Scientific). To generate expression clones, all entry clones were used in LR Clonase™ II (Thermo Fisher Scientific) reactions with corresponding expression vectors: pSAT5‐DEST‐c(175‐end)EYFP‐C1(B) and pSAT4‐DEST‐n(174)EYFP‐Cl for BiFC assay (Lee et al., 2012), pH7WGF2 and pB7WGR2 for fusion with GFP/RFP, respectively (Karimi et al., 2002).

### Plant cells transfection

For protein localization, expression clones of *PpTRB* genes in the pH7WGF2 vector containing the eGFP protein‐coding sequence were used (Karimi et al., 2002). The localization of PpTRB proteins was analyzed in *P. patens* protonema protoplasts, *A. thaliana* root protoplasts and *N. tabacum* leaves.

*Physcomitrium patens* protoplasts were prepared and transfected using a PEG‐based procedure as described in Liu and Vidali (2011) from 7‐day‐old protonemata grown on BCDAT agar medium. For GFP localization and BiFC analysis (see below), 10 μg of plasmid DNA for each construct was used. Protoplasts with expression clones encoding PpTRB fused to GFP (pH7WGF2) were co‐transfected with mRFP‐Fibrillarin 1 (Pih et al., 2000), which labels the nucleolus, to enable simultaneous visualization of subcellular localization.

*Arabidopsis thaliana* root protoplasts were isolated and transfected with 10 μg of plasmid DNA per construct using the PEG‐mediated method as described in Kolářová et al. (2021).

Transient heterologous expression in *N. tabacum* (SR1 Petit Havana) leaves was performed as described in Kusová et al. (2023). The leaf epidermal cells were transfected by the infiltration procedures described in Voinnet et al. (2000).

The fluorescence was observed using a laser scanning confocal microscope Zeiss LSM 880 with C‐Apochromat 63×/1.2 W Korr M27 objective using a 488 nm laser for GFP.

### Protein localization and FLIM‐FRET

For FLIM‐FRET analysis, coding sequences of PpTRB proteins fused to GFP/RFP (pH7WGF2/pB7WGR2) were used. *Agrobacterium tumefaciens* competent cells (strain GV3101) were transfected with corresponding expression clones and these plasmids were used for transient expression in *N. tabacum* (SR1 Petit Havana) leaves (Voinnet et al., 2000). The empty RFP construct under the control of the 35S promoter (pB7WGR2) served as the negative control. Laser scanning confocal imaging microscope Zeiss LSM 780 AxioObserver equipped with external In Tune laser (488–640 nm, <3 nm width, pulsed at 40 MHz, 1.5 mW) C‐Apochromat 63 × water objective, NA 1.2 was employed for confocal microscopy. HPM‐100‐40 Hybrid Detector from Becker and Hickl GmbH including Simple‐Tau 150N (Compact TCSPC system based on SPC‐150N) with DCC‐100 detector controller for photon counting was used for FLIM‐FRET data acquisition. Zen 2.3 light version from Zeiss was used for processing confocal images. The acquisition and analysis of FLIM data involved the utilization of SPCM 64 version 9.8. and SPCImage version 7.3 from Becker and Hickl GmbH, respectively. A multiexponential decay model was used for fitting.

### Bimolecular fluorescence complementation (BiFC)

To analyze protein–protein interactions by BiFC, the *P. patens* protoplasts were prepared and transformed with expression clones of *PpTRB* genes in pSAT4‐DEST‐n(174)EYFP−Cl (pE3136/nYFP) and pSAT5‐DEST‐c(175‐end)EYFP−C1(B) (pE3130/cYFP) vectors. To control PEG‐based transfection efficiency and to label cell nuclei, the protoplasts were co‐transfected with a plasmid expressing mRFP fused to the nuclear localization signal of the VirD2 protein (mRFP−VirD2NLS) (Citovsky et al., 2006). As a negative control, an empty construct containing a complementary fragment of YFP (either pSAT5‐DEST‐c(175‐end)EYFP‐C1(B) or pSAT4‐DEST‐n(174)EYFP‐C1) under the control of the 35S promoter, was used. The transformation was performed as described by Liu and Vidali (2011) with minor modifications. After the incubation period of protoplasts with PEG solution, the protoplasts were diluted with 3 ml of MMg solution instead of W5 solution, then centrifuged at 250 ***g*** for 5 min and finally resuspended in 600 μl of MMg solution. Transfected protoplasts were incubated in the light, at room temperature overnight. The next day, the fluorescence was observed using a laser scanning confocal microscope Zeiss LSM 880 with C‐Apochromat 63×/1.2 W Korr M27 objective using 488 nm laser for GFP, 514 nm laser for YFP and 594 nm laser for RFP. The images obtained were adjusted in the ZEN 2.5 lite Blue software.

### TRB association with telomeric regions

For fluorescence imaging, coding sequences of PpTRB proteins fused to RFP (pB7WGR2) and the three‐component constructs (called dCas9:2xMS2:GFP) encode dCas9 of *S. pyogenes*, an *Arabidopsis* telomere‐specific sgRNA with integrated aptamer sequences (2× MS2) and aptamer coat proteins fused to three copies of fluorescent proteins (tdMCP:GFP; GFP binding to MS2) (Khosravi et al., 2020), were used. *Agrobacterium tumefaciens* competent cells (strain GV3101) were transfected with corresponding expression clones and these plasmids were used for transient expression in *N. tabacum* (SR1 Petit Havana) leaves (Voinnet et al., 2000).

Fluorescence imaging was performed using a Leica STELLARIS 8 FALCON confocal laser scanning microscope (Leica Microsystems, Wetzlar, Germany), equipped with a white light laser (WLL) and highly sensitive HyD X detectors. The system was operated via Leica LAS X software. Imaging was performed using an HC PL APO CS2 63×/1.20 WATER immersion objective. Images were acquired at a resolution of 376 × 376 pixels, with a scan speed of 200 Hz and a zoom factor of 6. Excitation wavelengths were tuned as follows: GFP was excited at 488 nm, and RFP at 590 nm. Emission was detected using HyD X detectors, with detection windows set to 500–550 nm for GFP and 600–650 nm for RFP. Sequential line scanning was employed to prevent spectral crosstalk between the fluorophores. Confocal image data were processed using Leica LAS X software. Images were acquired in 8‐bit depth and exported without compression or scaling to preserve the original fluorescence intensity values. Quantitative fluorescence analysis was performed to determine the relative signal intensities of the GFP and RFP channels in plant leaf tissue. Regions of interest (ROIs) were manually defined over specific fluorescent structures within the tissue, and mean pixel intensities were measured for each channel. For each ROI, corresponding background ROIs were selected in non‐fluorescent areas. Each bar in the chart represents the mean fluorescence intensity of a single ROI, grouped by channel (GFP or RFP), allowing for direct visual comparison. All samples were imaged and analyzed under identical settings, and all quantitative assessments were performed using the same workflow to ensure consistency across the dataset.

### Terminal restriction fragment analysis (TRF)

To monitor telomere length dynamics over time, protonemal cultures were initiated from long‐term storage cultures of *P. patens* mutant lines. These long‐term storage cultures were prepared shortly after the establishment and verification of the mutant lines and maintained at 4°C under dim light, which permits long‐term storage without subculture. A small piece of stored tissue was homogenized in BCDAT medium and plated on BCDAT plates, designated as the first passage. The 7‐day‐old protonemal culture was scraped off, homogenized, and transferred to BCDAT fresh plates. From the third passage onward, material from a single plate was distributed to multiple plates, enabling both culture maintenance and collection of tissue for analysis. This subculture procedure was repeated weekly for 12–14 weeks. DNA for TRF analysis was isolated from protonema collected at weeks 2/3, 8/9, and 12–14, corresponding to cultures grown on 2–3 fully developed plates. Genomic DNA (gDNA) from WT and mutant lines was isolated according to Dellaporta et al. (1983). The quality of DNA was checked, and its concentration was determined by electrophoresis in a 1% (w/v) agarose gel stained with ethidium bromide using Gene Ruler 1 kb DNA Ladder (Thermo Scientific, Waltham, MA, USA) as standard and Multi Gauge software (Fujifilm, Tokyo, Japan).

Telomere lengths were analyzed as described in Fojtová et al. (2015). Samples of 1 μg genomic DNA were digested by 1 μl of MseI overnight (10 000 units ml^−1^, NEB). The next day, an additional 1 μl of MseI was added to samples and the digestion continued for about 4 h. Then, samples were separated in 1% (w/v) agarose gel followed by Southern blot on Amersham™ Hybond™ ‐ XL membrane (Ge Healthcare, Chicago, IL, USA). The membrane was hybridized overnight at 65°C (to avoid signals from interstitial telomeric sequences) with plant telomere probe. The plant telomere probe (TTTAGGG)_n_ was synthesized by non‐template PCR (IJdo et al., 1991; Závodník et al., 2023) and radioactively labeled with [^32^P‐dATP] using DecaLabel DNA labeling kit (Thermo Scientific). Signals were visualized using FLA7000 imager (Fujifilm).

Boxplot values were obtained by evaluating telomere length profiles of mutant and corresponding WT plants using the R‐based online toolset WALTER (Lyčka et al., 2021), using the default setup with background correction. For statistical analysis, median values generated by the IntensityAnalyser module of WALTER were subjected to a linear model followed by generalized linear hypothesis testing (GLHT; Hothorn et al., 2025), analogous to Dunnett's test with Benjamini‐Hochberg correction for multiple comparisons.

### Phylogenetic analysis

To effectively identify TRB orthologs across the Streptophyta clade, we deployed an iterative approach, wherein after each step, the query dataset was enriched with the newly discovered protein sequences. The resulting multiple sequence alignment (MSA) was then used to target lineages lacking positive hits. The iteration proceeded until all relevant data had been processed.

Previously discovered TRB sequences (Kusová et al., 2023) were queried against the NCBI nr database (MMseqs Version: 14.7e284) and restricted output to alignments that are within the 1.0E‐06 threshold *e*‐value with default sensitivity.

The retrieved Streptophyta protein sequences were aligned with FAMSA 2 (Deorowicz et al., 2016) and converted into a MMseqs2 profile. Relying on iterative profile‐to‐sequence search in MMseqs2, we probed additional taxa not included in nr. Taxa were selected to cover missing lineages in Streptophyta. In the case of annotated genomes, we used translated gene sequences (proteome); otherwise we selected taxa with available transcriptome data. We used publicly available RNA‐seq data for 30 species. To remove poor‐quality reads, polyAtails, and singletons, reads were filtered with fastp (Chen, 2023; Chen et al., 2018) running on the following settings: ‐‐trim_poly_g ‐‐trim_poly_x ‐‐low_complexity_filter ‐‐cut_tail. The filtered read sets were assembled using rnaSPAdes v4.0.0 with default options (Bushmanova et al., 2019). *De novo* assembled transcriptomes and previous assemblies from 1KP (One Thousand Plant Transcriptomes Initiative; Leebens‐Mack et al., 2019) were searched with MMseqs2. When medium sensitivity screening failed to retrieve any bona fide matches, we repeated the search in high sensitivity mode (‐s 7.5). Protein‐coding open reading frames were identified from the profile alignments. For annotated genomes lacking protein sequences, we obtained the proteome from CDS extraction with gffread (Pertea & Pertea, 2020) and translation with orfipy release 0.0.4 (Singh & Wurtele, 2021) keeping the longest open reading frames (ORFs).

When no positive hits were recovered from transcriptome nor proteome data, we turned our attention to readily available unannotated genomes. We defined TRB gene candidate regions with two complementary approaches: (1) using miniprot (Li, 2023) for mapping previously identified proteins to the genomes and improved exon delimitation with Miniprot boundary scorer (https://github.com/tomasbruna/miniprot‐boundary‐scorer) (Brůna et al., 2023), (2) the MSA from the recovered proteins was used to build profile hidden Markov models for BATH (Krause et al., 2024) and genomes were probed with the bathsearch module. We retained genes that were validated by the highest scoring predictions from both approaches and translated them with orfipy. Transcriptomes, proteomes, and genomes were assessed for completeness by running BUSCO v 5.8.0 in the appropriate mode against the Viridiplantae databases (viridiplantae_odb10) (Seppey et al., 2019). All newly identified protein sequences were checked against the InterPro 103.0 database (Apweiler et al., 2001) for the presence of Myb‐like, H1/5‐like and coiled‐coil domains in an N to C‐terminal orientation.

As the broad evolutionary patterns of TRB evolution have been previously studied in Angiospermae (Kusová et al., 2023), the MSA was downsized for phylogenetic purposes to a single taxon per family in this clade. We kept taxa with the highest TRB copy number and retained all *Brassicaceae* sequences to get a deeper insight into the gene diversification rate within *A. thaliana* relatives.

ModelFinder (Kalyaanamoorthy et al., 2017) was used to determine the best fitting molecular evolutionary models for maximum likelihood analyses. Protein mixture and non‐reversible models (Minh et al., 2021) were tested alongside the base time‐reversible amino acid models. Selected models were used for building the phylogenies in the IQ‐TREE software package (Nguyen et al., 2015); branch support was assessed with ultrafast bootstrap approximation with 10 000 replicates (Hoang et al., 2018). ModelFinder recovered that the reversible JTT+I+R8 protein evolution model was a better fit than the LG+C10+F+I+G mixture model that was ranked a close second according to the Bayesian Information Criterion (164 429.949 versus 164 435.425). We attempted to infer the most likely root placement using the non‐reversible unrestricted model (UNREST) in IQ‐TREE. Because of low *rootstrap* support values (21.4%), the root location was unresolved and consequently we rooted the tree with the Chlorokybophyceae lineage as out‐group, in accordance with the accepted phylogeny of Streptophyta. Since out‐group rooting might distort in‐group topology due to long‐branch attraction where distantly related or fast‐evolving taxa spuriously branch with the out‐group (Philippe et al., 2011), we compared the topology with the tree obtained from a reduced dataset without out‐group sequences. The two topologies were perfectly congruent, thus dismissing a reconstruction error arising from distant out‐group inclusion. Trees were visualized in iTOL v6 (Letunic & Bork, 2024).

## ACCESSION NUMBERS

PpTRB1 (Pp3c13_1060V3.1, Pp6c13_440); PpTRB2 (Pp3c13_1310V3.1, Pp6c13_610); PpTRB3 (Pp3c3_1630V3.1, Pp6c3_810).

## AUTHOR CONTRIBUTIONS

PPS and JF conceived the study. ES and AK performed the cloning; AK performed localization and protein–protein interaction studies. Telomere measurements were performed by AK with the help of IGP, and RNA‐seq analysis was carried out by AK with the help of JR. FLIM‐FRET was performed by JS with the help of AK and JH. TPM calculations for GOLEM were done by BK, with the help of DH. TP and AK helped with GOLEM program analysis. ML performed GO analysis. JR performed statistical analysis. The mutant plants were established by MH, with the help of DZ. The evolution study was performed by YJKB. PPS wrote the paper with the help of all co‐authors.

## CONFLICT OF INTEREST

The authors report no declarations of interest.

## Supporting information


**Figure S1.** Schematic representation of mutant lines.
**Figure S2.** Representative images of all verified mutant lines, prepared as described in the Methods section.
**Figure S3.** Validation of disrupted gene transcription in *pptrb* lines using QuantSeq.
**Figure S4.** The transition of chloronema to caulonema in the dark.
**Figure S5.** Differential gene expression in *pptrb* mutants.
**Figure S6.** Volcano plots of differentially expressed genes between WT and *pptrb* single and double mutant lines.
**Figure S7.** MA plots of differentially expressed genes between WT and *pptrb* single and double mutant lines.
**Figure S8.** Gene ontology (GO) enrichment analysis of differentially expressed genes in *pptrb* mutant lines.
**Figure S9.** Analysis of RNA‐seq data from single and double *pptrb* mutant lines in GOLEM web‐tool for the presence of *cis*‐regulatory motifs.
**Figure S10.** Telomere length analysis by TRF assay in single and double *pptrb* mutant lines across various mutant lines.
**Figure S11.** PpTRB proteins localize to the nucleus and nucleolus.
**Figure S12.** PpTRBs mutually interact in the nucleus.
**Figure S13.** PpTRBs mutually interact in the cytoplasm.
**Figure S14.** Maximum intensity projections and *z*‐stacks of PpTRB protein interactions in the cytoplasm.
**Figure S15.** PpTRBs nuclear speckles locate at same spots in nucleoplasm.
**Figure S16.** Calculated FRET efficiency from speckles and nucleoplasm for PpTRB interactions.
**Figure S17.** Phylogenetic analysis of TRB proteins across streptophyte taxa.

**Table S1.** List of differentially expressed genes (DEGs) mapped to Ppatens_318_v3.3 and Ppatens_870_v6.1.

**Table S2.** (a) List of genes from *P. patens* with transcripts per million (TPM) in WT and mutant plants, calculated using the pipeline described in Nevosád et al. (2025). (b) List of genes highly expressed (20% percentile; Nevosád et al., 2025) in WT and *pptrb* single and double mutants with *telo*‐box motifs ±100 bp from TSS.

**Table S3.** Summary of the transcriptomes, proteomes, and genomes analyzed.

**Table S4.** List of primers.

## Data Availability

The data that support the findings of this study are openly available in The European Nucleotide Archive (ENA) at https://www.ebi.ac.uk/ena/browser/view/PRJEB90481, reference number PRJEB90481.
