# Supplementary material for: TRB proteins in moss reveal their evolutionarily conserved roles in plant development and telomere maintenance
Source: Plant J. 2025 Nov 14;124(3):e70574. doi: 10.1111/tpj.70574 (PMC12616469; doi:10.1111/tpj.70574)
Supplement: Supplementary file 1 — Figure S1. Schematic representation of mutant lines. Figure S2. Representative images of all verified mutant lines, prepared as described in the Methods section. Figure S3. Validation of disrupted gene transcription in pptrb lines using QuantSeq. Figure S4. The transition of chloronema to caulonema in the dark. Figure S5. Differential gene expression in pptrb mutants. Figure S6. Volcano plots of differentially expressed genes between WT and pptrb single and double mutant lines. Figure S7. MA plots of differentially expressed genes between WT and pptrb single and double mutant lines. Figure S8. Gene ontology (GO) enrichment analysis of differentially expressed genes in pptrb mutant lines. Figure S9. Analysis of RNA‐seq data from single and double pptrb mutant lines in GOLEM web‐tool for the presence of cis‐regulatory motifs. Figure S10. Telomere length analysis by TRF assay in single and double pptrb mutant lines across various mutant lines. Figure S11. PpTRB proteins localize to the nucleus and nucleolus. Figure S12. PpTRBs mutually interact in the nucleus. Figure S13. PpTRBs mutually interact in the cytoplasm. Figure S14. Maximum intensity projections and z‐stacks of PpTRB protein interactions in the cytoplasm. Figure S15. PpTRBs nuclear speckles locate at same spots in nucleoplasm. Figure S16. Calculated FRET efficiency from speckles and nucleoplasm for PpTRB interactions. Figure S17. Phylogenetic analysis of TRB proteins across streptophyte taxa. [file TPJ-124-0-s005.zip › Supplementary_Figures_minor_revisions.pdf]

TRB Proteins in Moss Reveal Evolutionarily Conserved Roles in Plant Development and Telomere Maintenance

Alžbeta Kusová[1,2], Marcela Holá[3], Ivana Goffová Petrová[1], Jiří Rudolf[1,2], Dagmar Zachová[1,2], Jan Skalák[1,2], Jan Hejátko[1,2], Božena Klodová[3], Tereza Přerovská[1,2], Martin Lyčka[1,2], Eva Sýkorová[4], Yann J. K. Bertrand[5], Jiří Fajkus[1,2], David Honys[3], and Petra Procházková Schrumpfová[1,2] *

[1]Laboratory of Functional Genomics and Proteomics, National Centre for Biomolecular Research, Faculty of Science, Masaryk University, Brno, Czech Republic

[2]Mendel Centre for Plant Genomics and Proteomics, Central European Institute of Technology, Masaryk University, Brno, Czech Republic

[3]Institute of Experimental Botany of the Czech Academy of Sciences, Prague, Czech Republic

[4]Institute of Biophysics of the Czech Academy of Sciences, Brno, Czech Republic

[5]Institute of Botany of the of the Czech Academy of Sciences, Průhonice, Czech Republic

Supplementary Figures

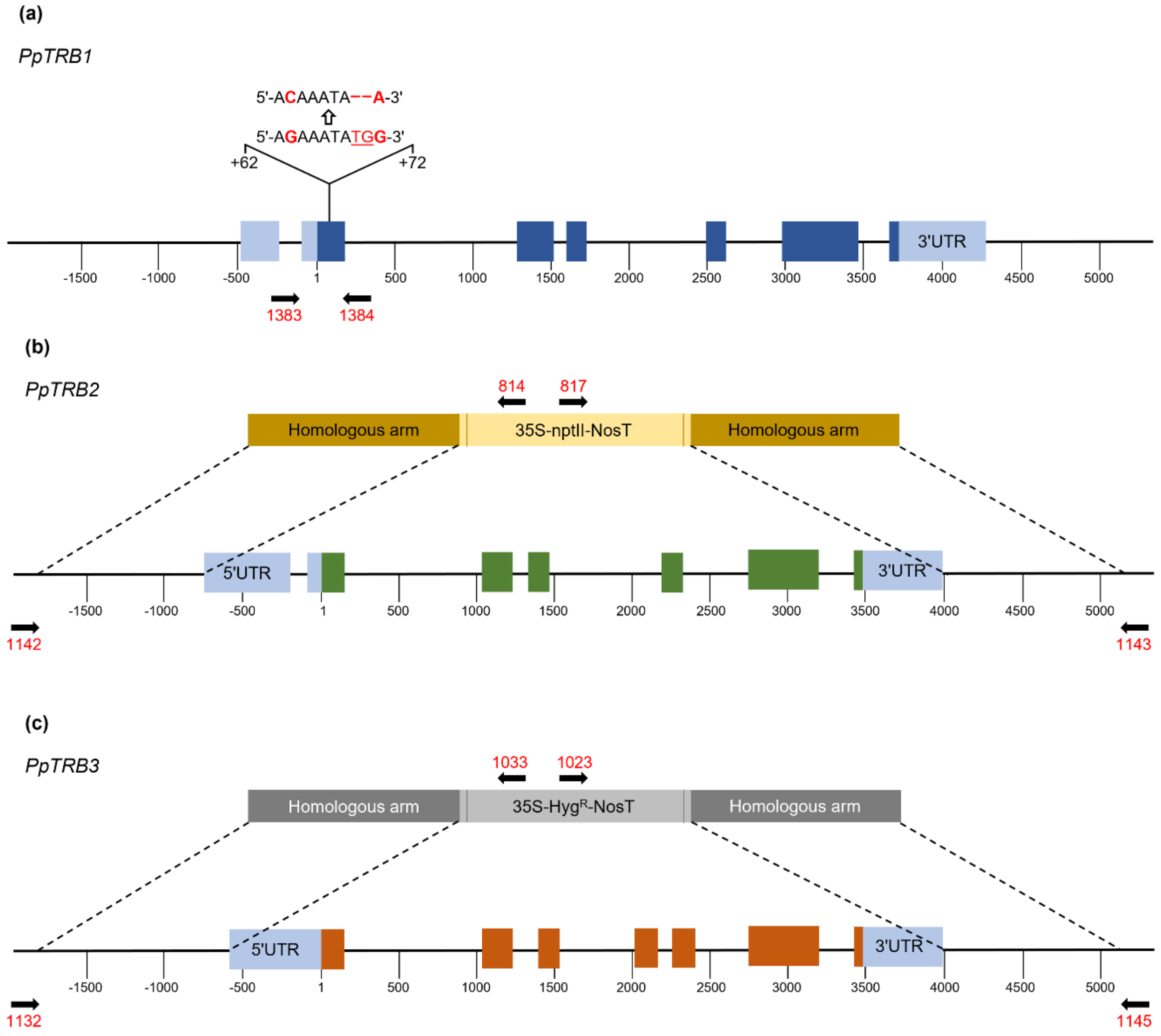

Supplementary Figure 1. Schematic representation of mutant lines. (a) Diagram of *PpTRB1* locus indicating the site of incorporated mutations. Nucleotide positions are given relative to the ATG codon, with adenine designated as position +1. Diagrams of *PpTRB2* **(b)** and *PpTRB3* **(c)** loci showing the locations of knockout cassettes containing homologous arms flanking the selectable marker gene, along with positions of genotyping primers. Notably, non-specific transcriptional upregulation downstream of the 35S-HygR-NosT selection cassette — used for homologous recombination-based mutagenesis — was observed in *pptrb2* and *pptrb3* lines.

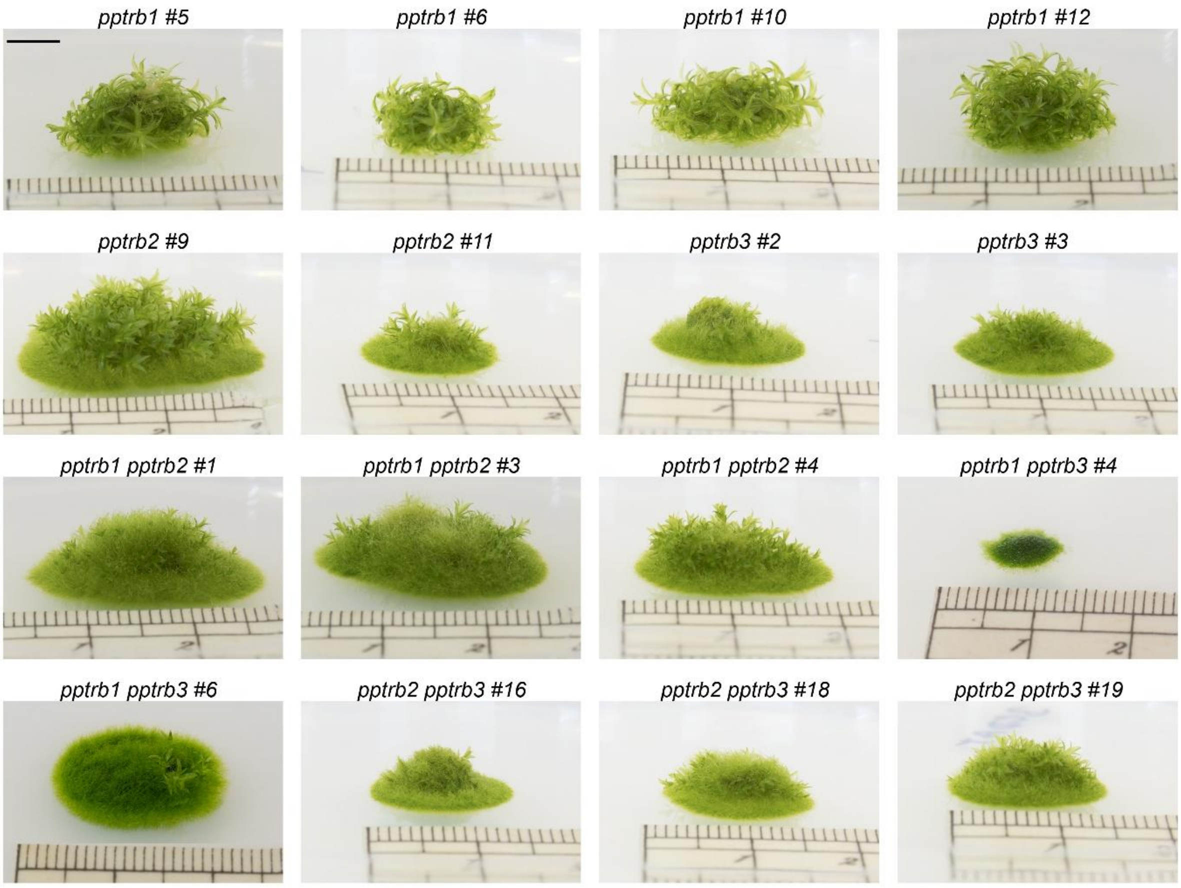

Supplementary Figure 2. Representative images of all verified mutant lines, prepared as described in the Methods section. For the mutant analysis, multiple lines were generated for each representative mutant (single and double). Single representative line was selected for further investigation and is also depicted in Figure 1: *pptrb1 #5*, *pptrb2 #11*, *pptrb3 #2*, *pptrb1 pptrb2 #3*, *pptrb1 pptrb3 #6* and *pptrb2 pptrb3 #16*.

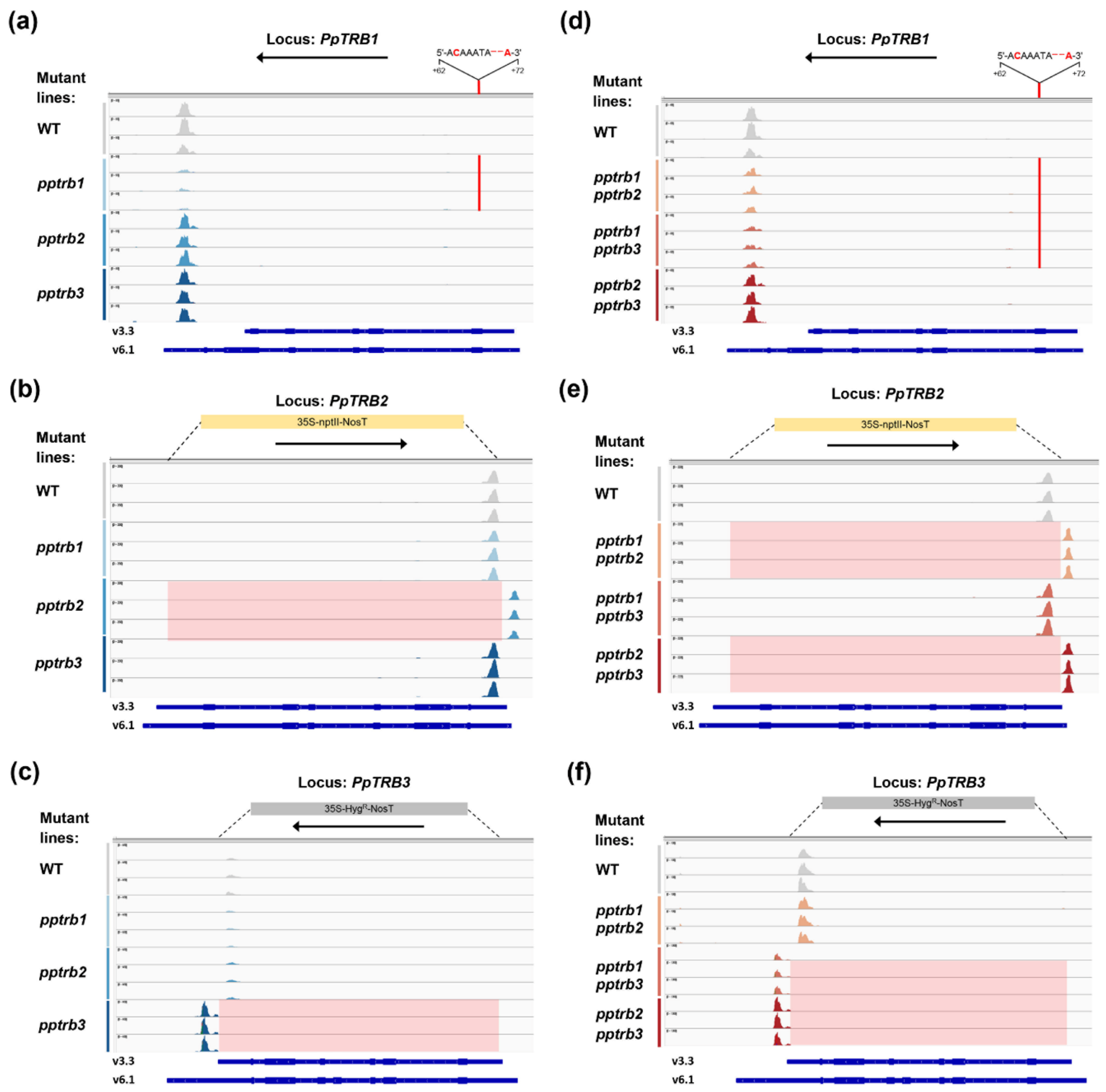

Supplementary Figure 3. Validation of disrupted gene transcription in *pptrb* lines using QuantSeq. RNA-Seq QuantSeq, which selectively captures the 3' ends of polyadenylated transcripts, was employed to assess transcript abundance in *PpTRB1*, *PpTRB2*, and *PpTRB3* loci. Sequencing reads were aligned to the corresponding genomic regions, and the results were visualized using the Integrative Genomics Viewer (IGV) with both v3.3 and v6.1 genome annotations. Schematic diagrams of the mutations and deletions characterizing each *pptrb* mutant line are presented alongside the mapped read profiles. IGV peak patterns revealed a clear knock-down of *PpTRB1* expression in *pptrb1* mutants and complete knock-out of *PpTRB2* and *PpTRB3* expression in *pptrb2* and *pptrb3* mutant lines, respectively.

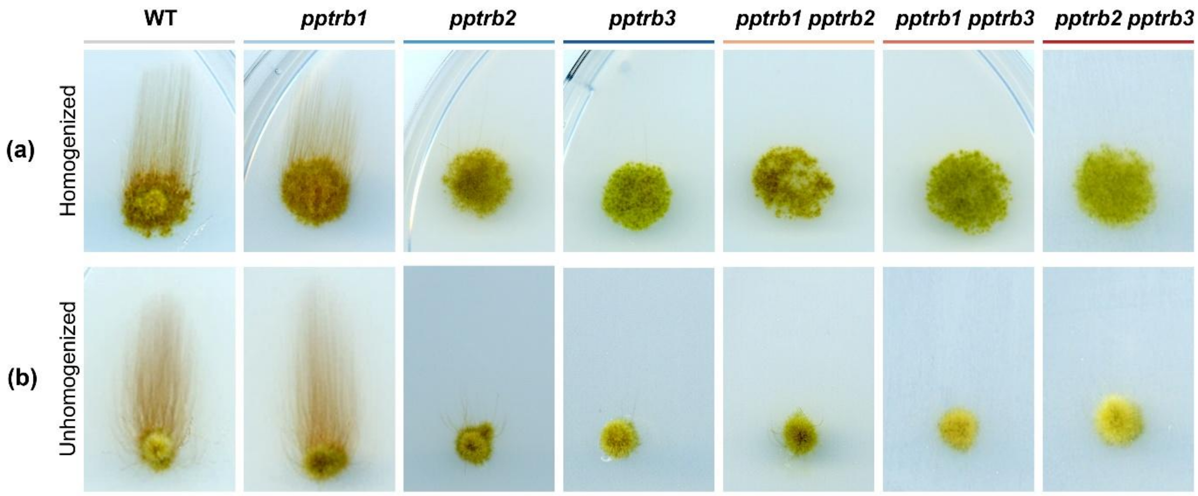

Supplementary Figure 4. The transition of chloronema to caulonema in the dark. Mosses grown under light conditions for one week were transferred to darkness for three weeks. Under these conditions, WT plants developed long, negatively gravitropic caulonemata, while *pptrb2*, *pptrb3*, and all double mutants exhibited a significant reduction in caulonemal growth. **(a)** A 50 µl aliquot of the homogenized protonema, collected during the mosses' passaging, was spotted onto the plate. **(b)** A small piece (approximately 1 mm²) from a one-week-old colony was transferred onto the plate.

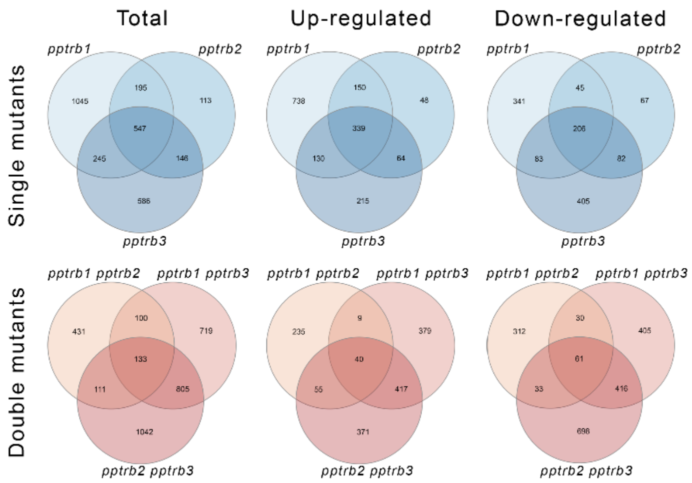

Supplementary Figure 5. Differential gene expression in *pptrb* mutants. Venn diagram illustrating the overlap of differentially expressed genes (DEGs) among *pptrb* single and double mutants using the reference genome v6.1. The total number of DEGs, as well as the subsets of up-regulated and down-regulated genes, are indicated.

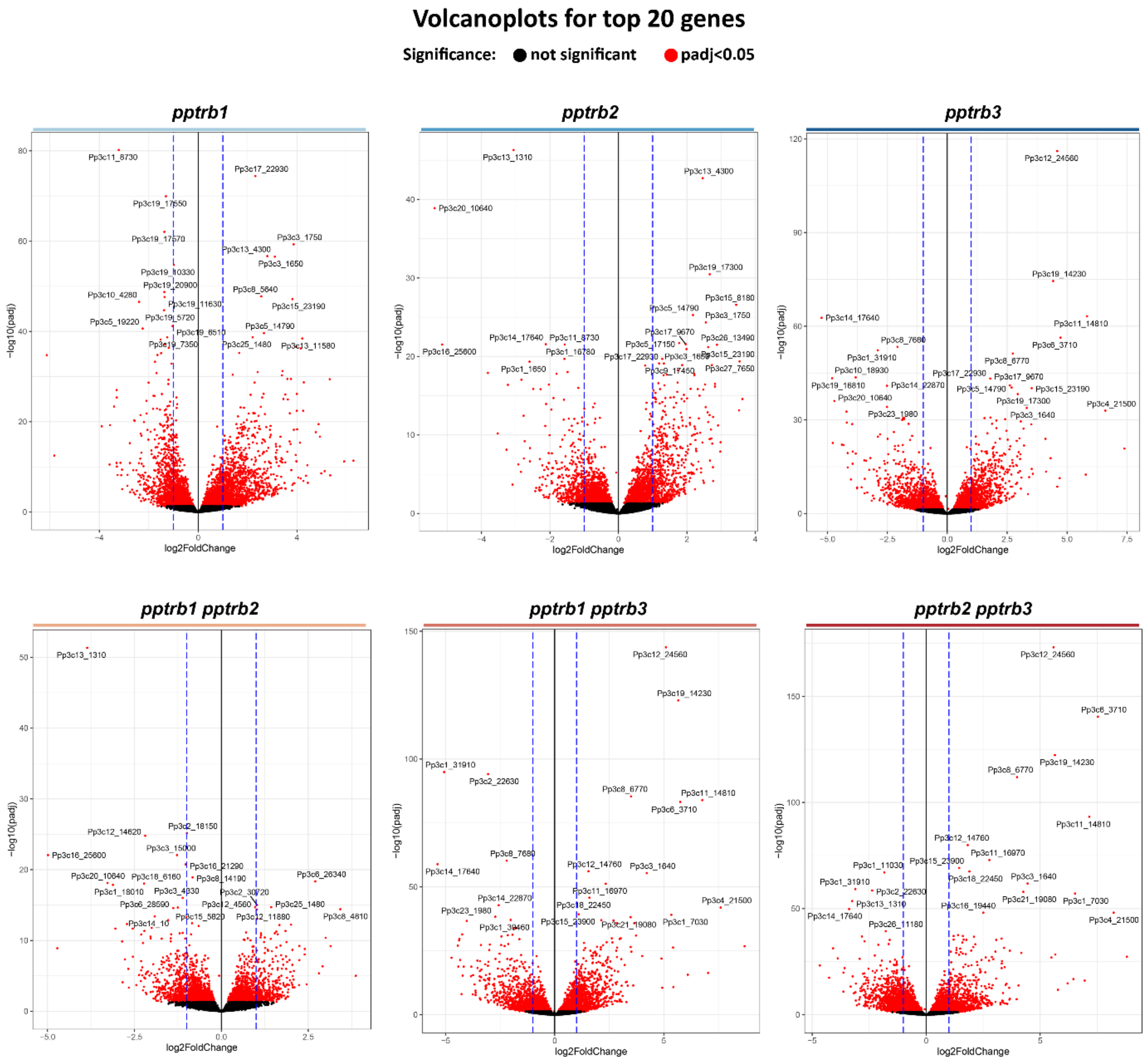

Supplementary Figure 6. Volcano plots of differentially expressed genes between WT and *pptrb* single and double mutant lines. Volcano plots illustrate the differential gene expression profiles between wild-type (WT) and *pptrb* mutant lines using the reference genome v3.3. The top 20 differentially expressed genes are labeled with their corresponding *Pp* gene identifiers. Genes were considered significantly differentially expressed if they met the thresholds of $p < 0.05$ and absolute fold change > 1. Red dots represent significantly altered genes, while black dots denote genes that do not meet the significance criteria.

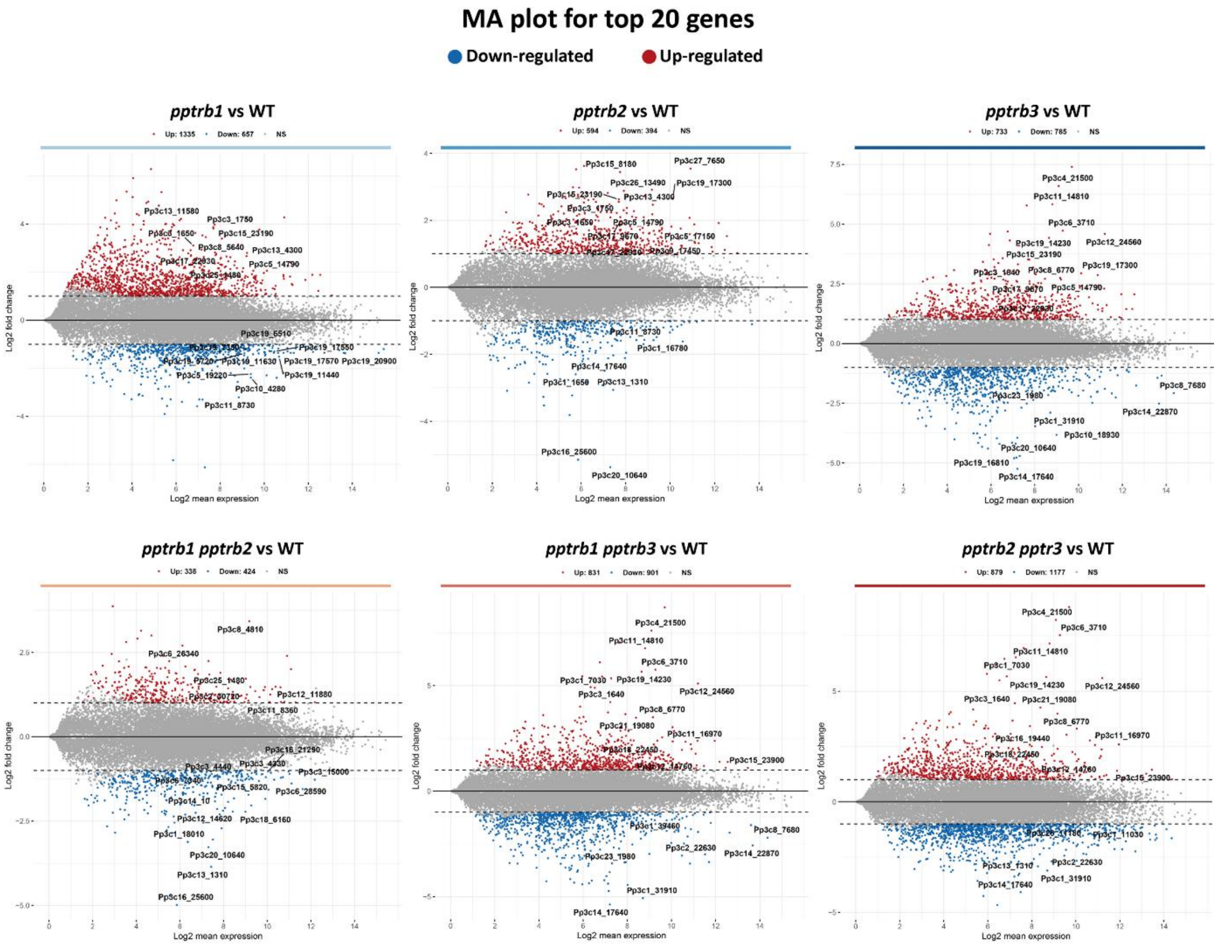

Supplementary Figure 7. MA plots of differentially expressed genes between WT and *pptrb* single and double mutant lines. MA plots display the log_2 fold changes in gene expression (y-axis) plotted against the mean expression levels (x-axis) for comparisons between wild-type (WT) and *pptrb* mutant lines using the reference genome v3.3. The top 20 differentially expressed genes are highlighted and labeled with their respective *Pp* gene identifiers.

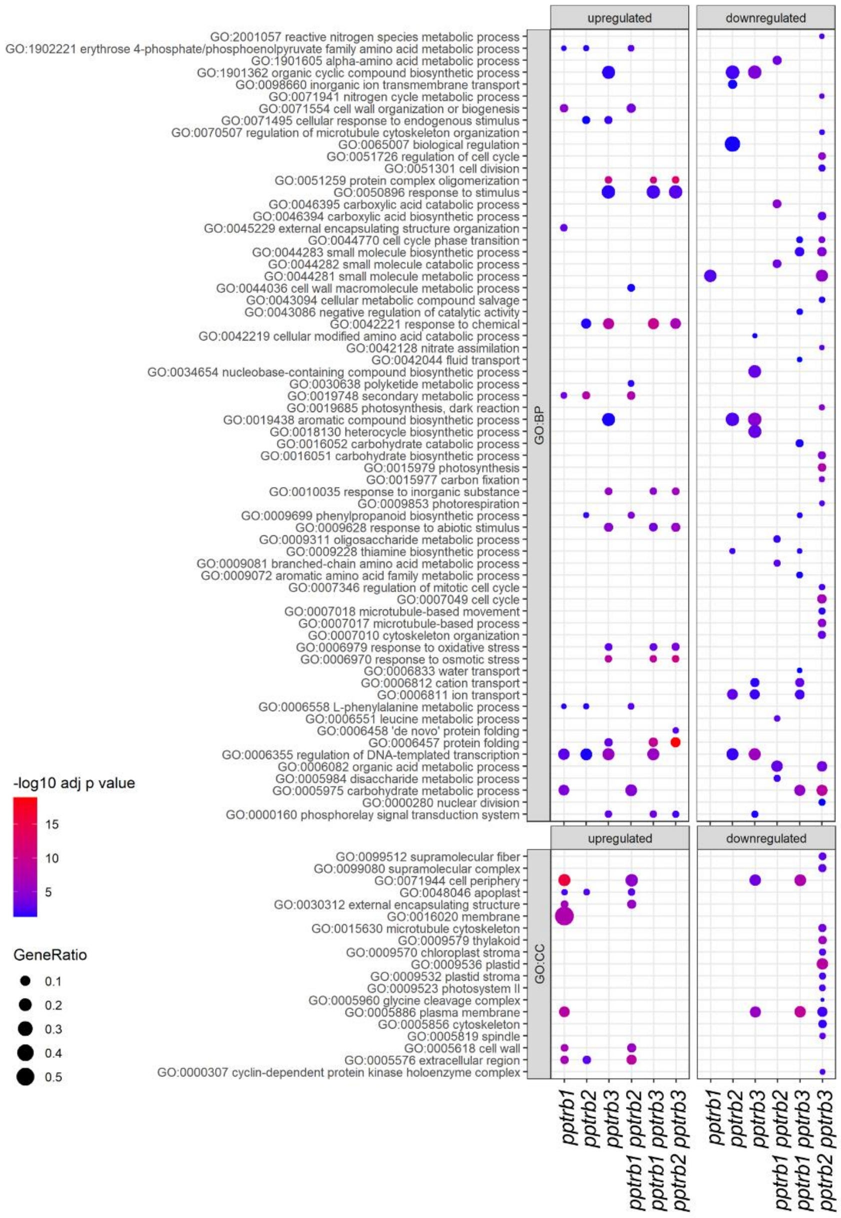

Supplementary Figure 8. Gene Ontology (GO) enrichment analysis of differentially expressed genes in *pptrb* mutant lines. GO enrichment analysis was performed on differentially expressed genes (DEGs) using g:Profiler. To reduce redundancy, the resulting GO terms were filtered using REVIGO with a similarity cutoff value of *C* = 0.5. Adjusted *p*-values for each GO term were obtained from g:Profiler. The GeneRatio represents the proportion of DEGs annotated to a given GO term and was calculated as the ratio of *intersection_size* to *query_size*, as defined by g:Profiler.

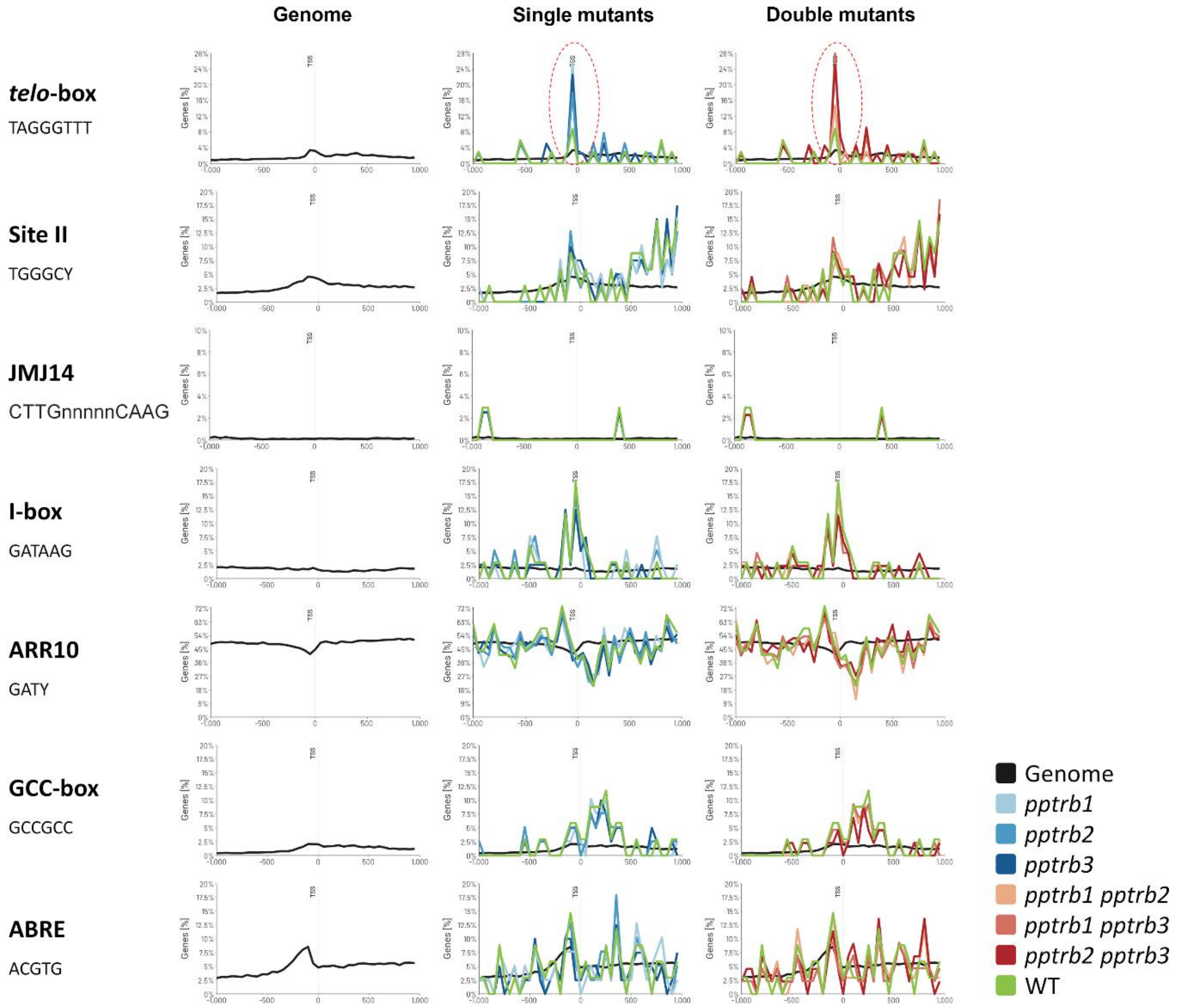

Supplementary Figure 9. Analysis of RNAseq data from single and double *pptrb* mutant lines in GOLEM web-tool for the presence of *cis*-regulatory motifs. The GOLEM tool (Nevosád *et al.*, 2025) was used to analyze RNA-Seq data from wild-type (WT) and *pptrb* mutant plants for the presence and positional distribution of cis-regulatory motifs relative to the transcription start site (TSS). The analysis revealed several regulatory motifs in the promoter regions of genes with the highest expression levels in *pptrb* mutants. Notably, the *telo*-box motif was more frequently observed in promoters of highly expressed genes in *pptrb1, pptrb3, pptrb1 pptrb3 and pptrb2 pptrb3* mutants compared to WT (GLMM, negative binomial distribution, emmeans post-hoc, Benjamini Hochberg correction). In contrast, the JMJ14 and Site II motifs—previously linked to TRB protein function—did not show such difference in *pptrb* mutants when compared to WT. Analyses were performed using the *P. patens* reference genome v3.3. The detailed procedure for statistical analysis can be found in the main text.

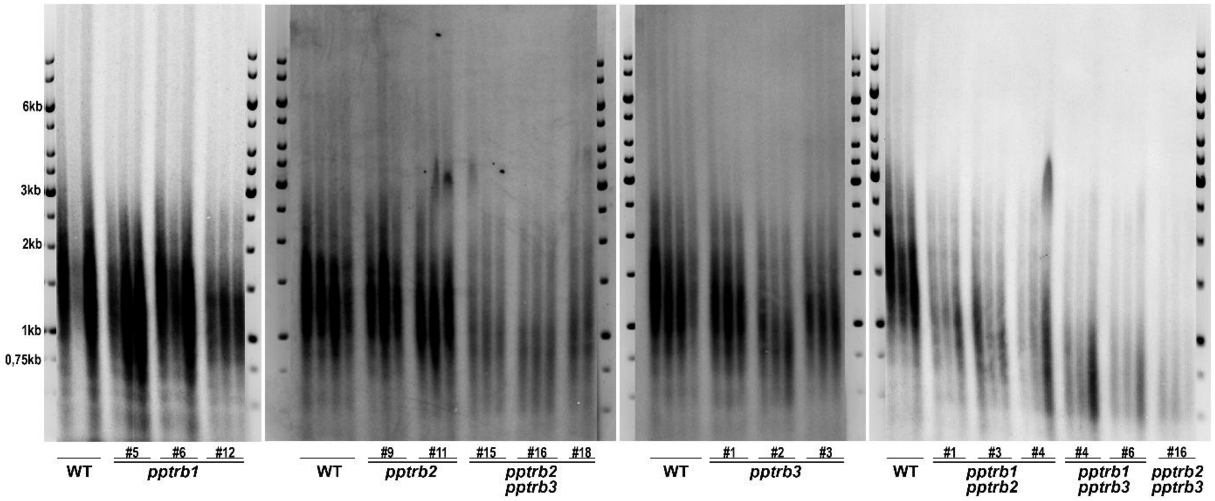

Supplementary Figure 10. Telomere length analysis by TRF assay in single and double *pptrb* mutant lines across various mutant lines. DNA was extracted at different passage and subjected to terminal restriction fragment (TRF) analysis. DNA molecular weight markers (kb) are shown.

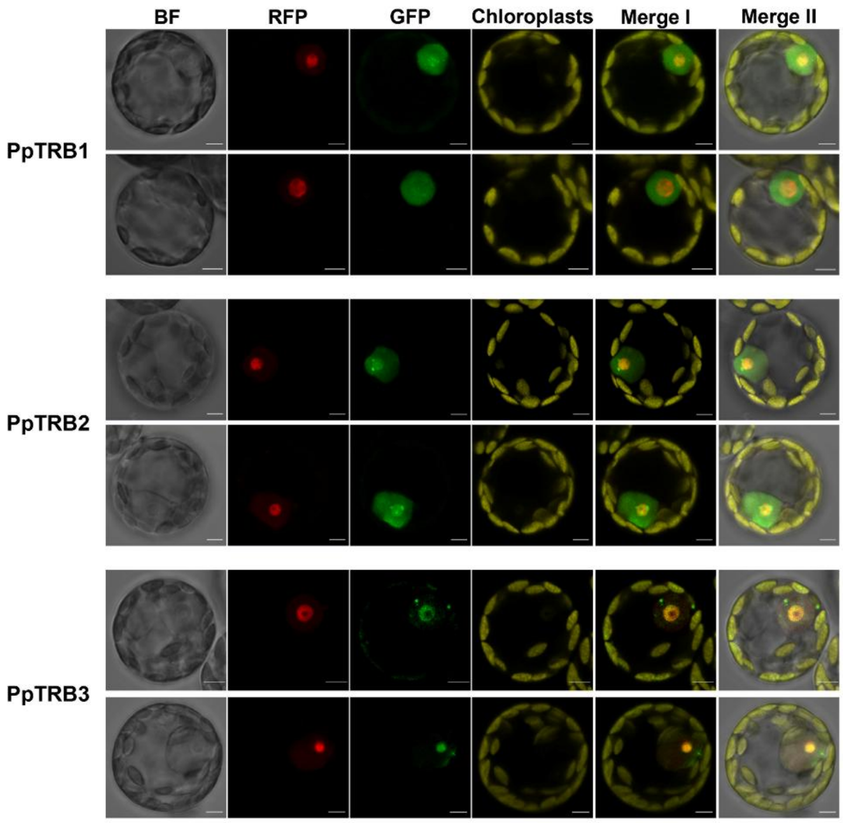

Supplementary Figure 11. PpTRB proteins localize to the nucleus and nucleolus. PpTRB proteins were fused with GFP and transfected into *P. patens* protoplasts together with mRFP-Fibrillarin, which labels the nucleolus. PpTRB1 and PpTRB2 proteins localize to the nucleus, while PpTRB3 localizes only to the nucleolus. Individual channels representing Bright Field (BF), RFP, GFP and chloroplast autofluorescence are shown. *Merge I* displays the merged channels of RFP, YFP, and chloroplast autofluorescence. *Merge II* displays the merged channels of BF, RFP, YFP, and chloroplast autofluorescence. Scale bars = 5 µm.

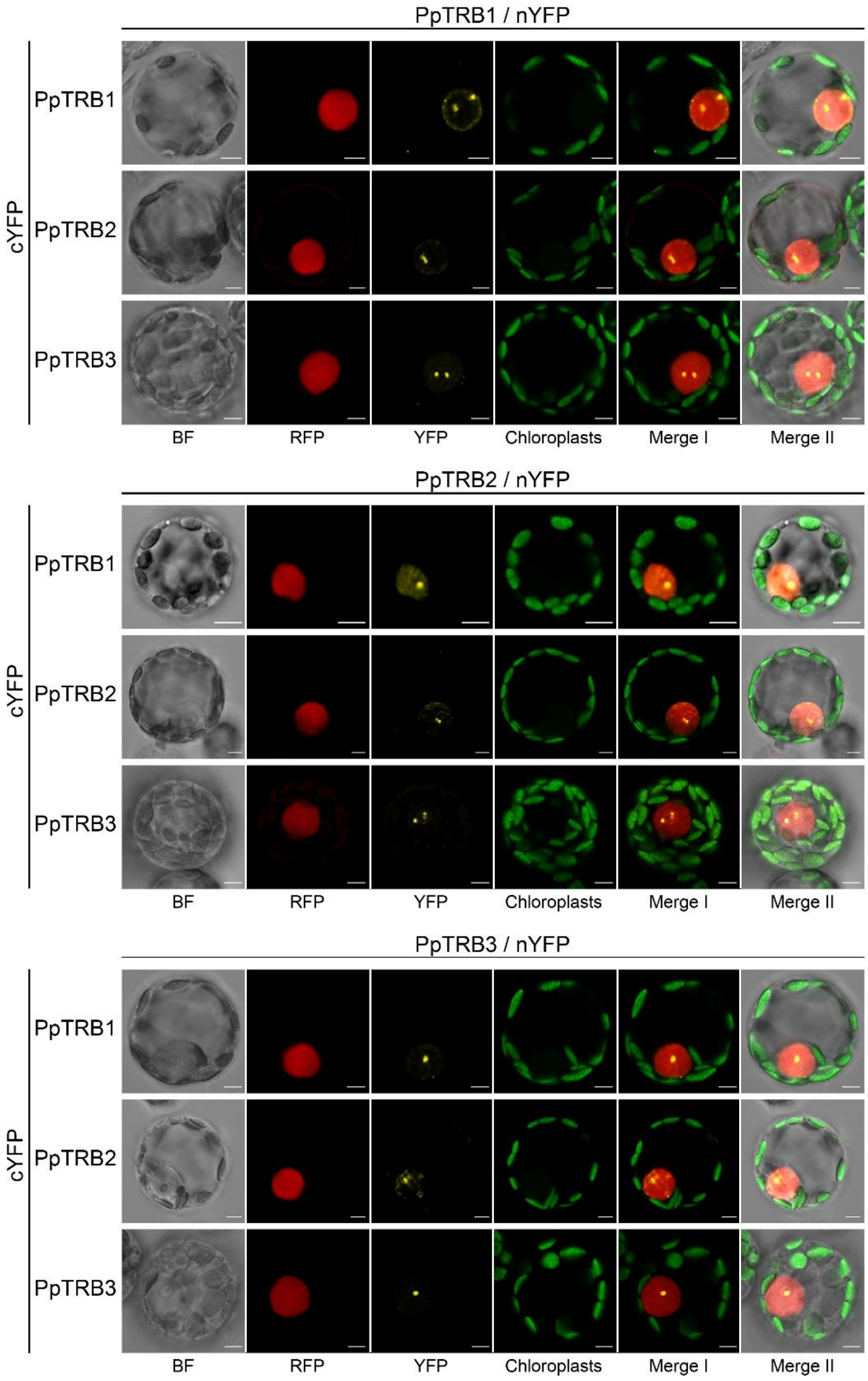

Supplementary Figure 12. PpTRBs mutually interact in the nucleus. Protein–protein interactions among *PpTRB* proteins were analysed using a Bimolecular Fluorescence Complementation (BiFC) assay. *PpTRB* constructs fused to either the N-terminal (nYFP) or C-terminal (cYFP) fragments of YFP were co-expressed in protoplasts derived from 7-day-old *P. patens* protonema. Fluorescence signals were visualized in individual channels. YFP channel images reveal distinct fluorescent foci localized within the nucleus, indicating physical interactions between *PpTRB* proteins. *Merge I* displays a merged channels of RFP, YFP, and chloroplast autofluorescence. *Merge II* includes bright-field (BF) in addition to RFP, YFP, and chloroplast autofluorescence channels. Scale bars = 5 µm.

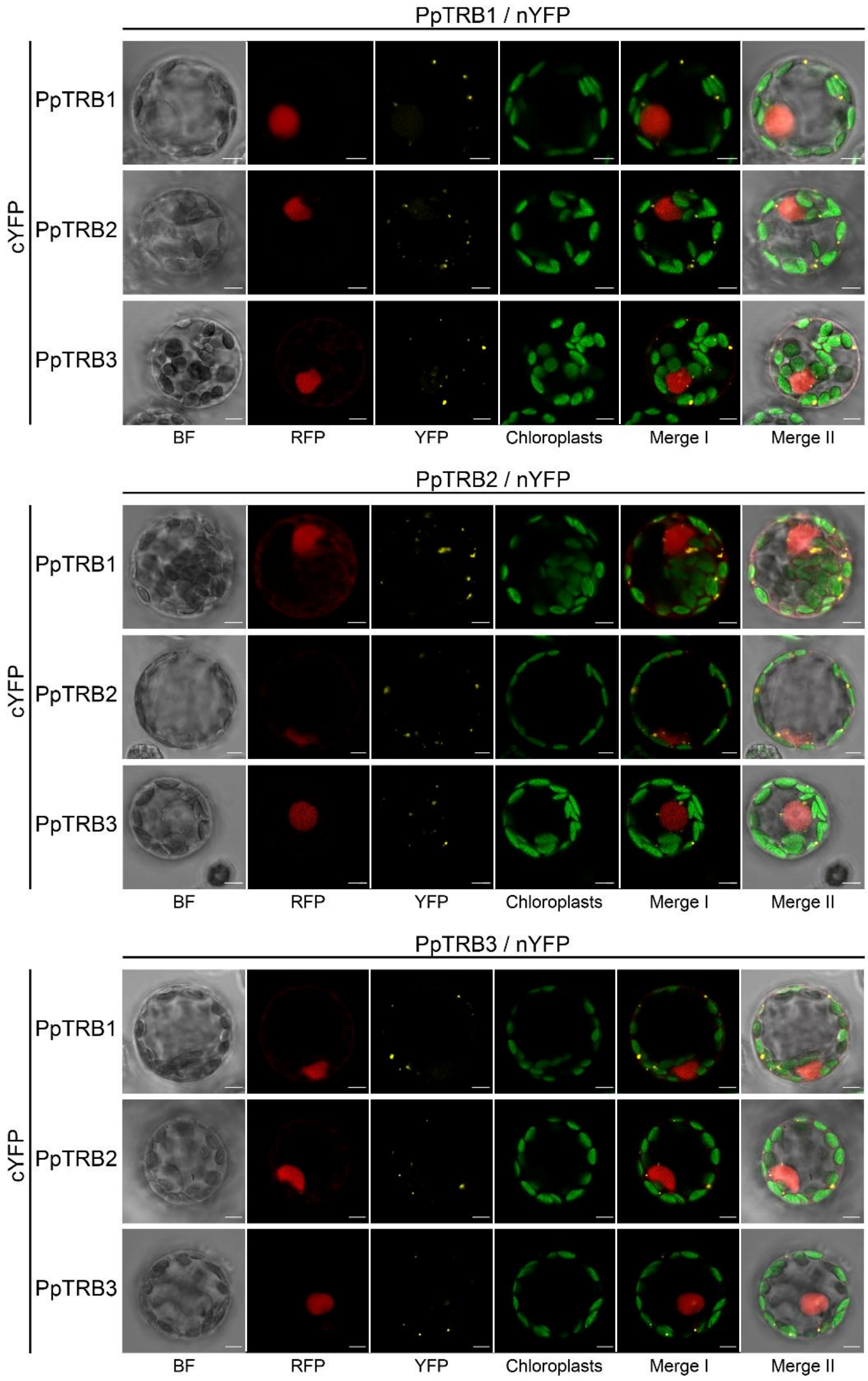

Supplementary Figure 13. PpTRBs mutually interact in the cytoplasm. Protein–protein interactions among *PpTRB* proteins were investigated using a Bimolecular Fluorescence Complementation (BiFC) assay. Constructs encoding *PpTRBs* fused to either the N-terminal (nYFP) or C-terminal (cYFP) fragments of YFP were co-expressed in protoplasts isolated from 7-day-old *P. patens* protonema. Fluorescence signals are shown as individual channels. In the YFP channel, fluorescent foci are observed in the cytoplasm, indicating interactions occurring outside the nucleus. *Merge I* shows the overlay of RFP, YFP, and chloroplast autofluorescence signals. *Merge II* includes bright-field (BF) along with RFP, YFP, and chloroplast autofluorescence. Scale bars = 5 µm.

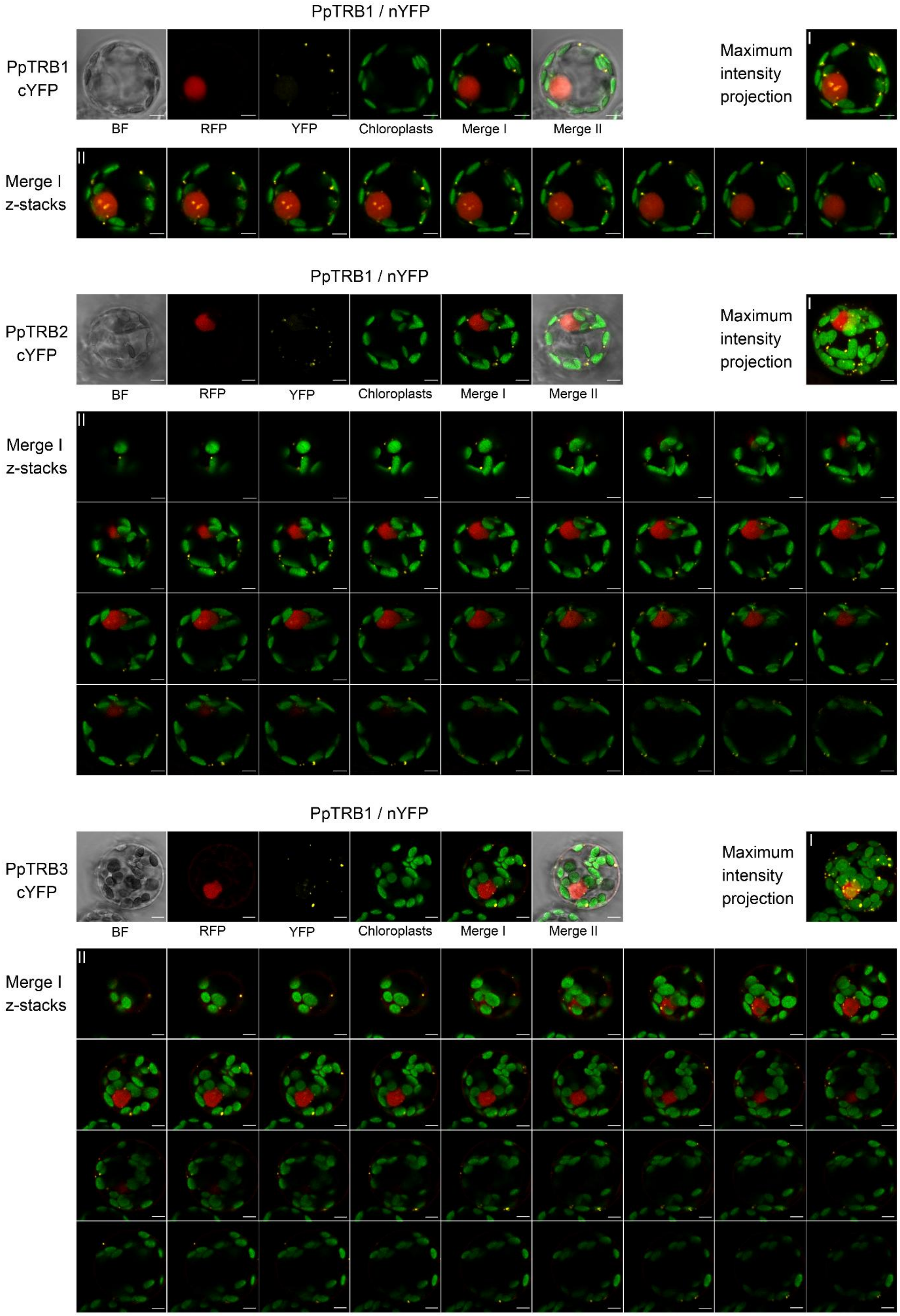
PpTRB1 / nYFP
PpTRB1
cYFP
BF
RFP
YFP
Chloroplasts
Merge I
Merge II
Maximum
intensity
projection
I
Merge I
z-stacks
II
PpTRB1 / nYFP
PpTRB2
cYFP
PpTRB1 / nYFP
PpTRB3
cYFP

Supplementary Figure 14. Maximum intensity projections and z-stacks of PpTRB protein interactions in the cytoplasm. Protein–protein interactions among PpTRB proteins were examined using BiFC in *P. patens* protoplasts derived from 7-day-old protonema cells and visualized by confocal microscopy. Panels show (I) maximum intensity projections of complete z-stacks and (II) representative single-plane sections of protoplasts (PpTRB1 with PpTRB1 – 0.52 µm each z-step size; PpTRB1 with PpTRB2 – 0.47 µm each z-step size; PpTRB1 with PpTRB3 0.47 µm each z-step size), scale bars = 5 µm.

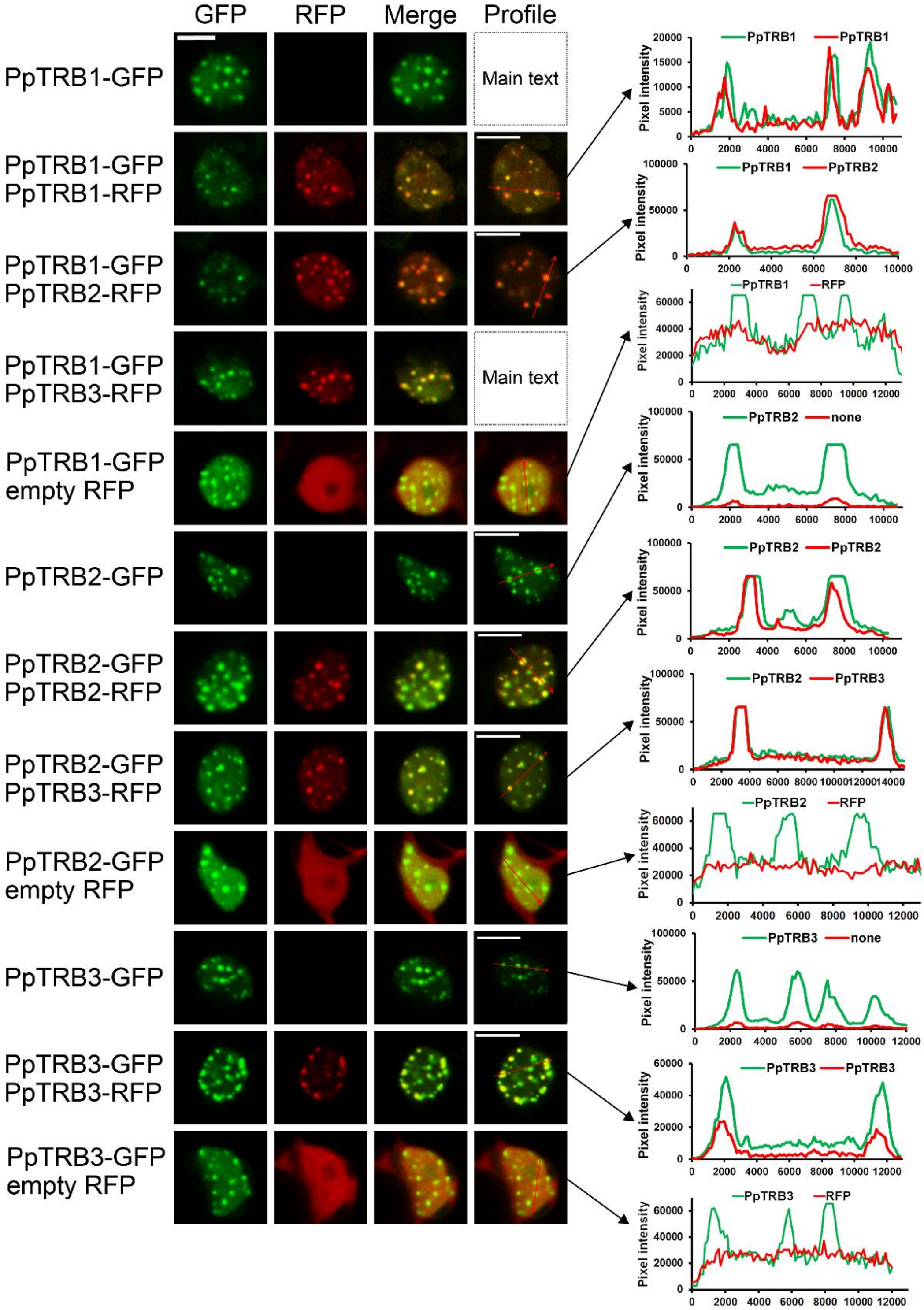

Supplementary Figure 15. PpTRBs nuclear speckles locate at same spots in nucleoplasm. Representative confocal microscopy images show the subnuclear localization of *PpTRB* proteins fused to GFP or RFP. Merged images reveal co-localization of *PpTRBs* at discrete nuclear foci. Scale bars = 5 µm. Intensity profiles corresponding to regions of interest (indicated by red arrows) confirm overlapping fluorescence signals, indicating co-localization of *PpTRBs* in nuclear speckles.

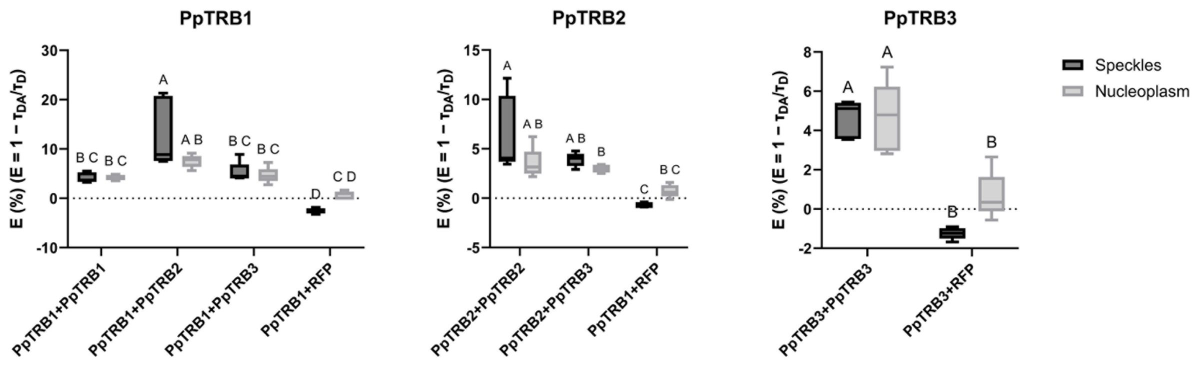

Supplementary Figure 16. Calculated FRET efficiency from speckles and nucleoplasm for PpTRB interactions. FRET efficiency E (%) was calculated as $E=(1-\tau_{DA}/\tau_D)\times100\%$, where τ_{DA} is the donor fluorescence lifetime in the presence of the acceptor, and τ_D is the donor lifetime in the absence of the acceptor. Statistical differences were determined by two-way ANOVA with Tukey's multiple comparison test ($P < 0.05$).

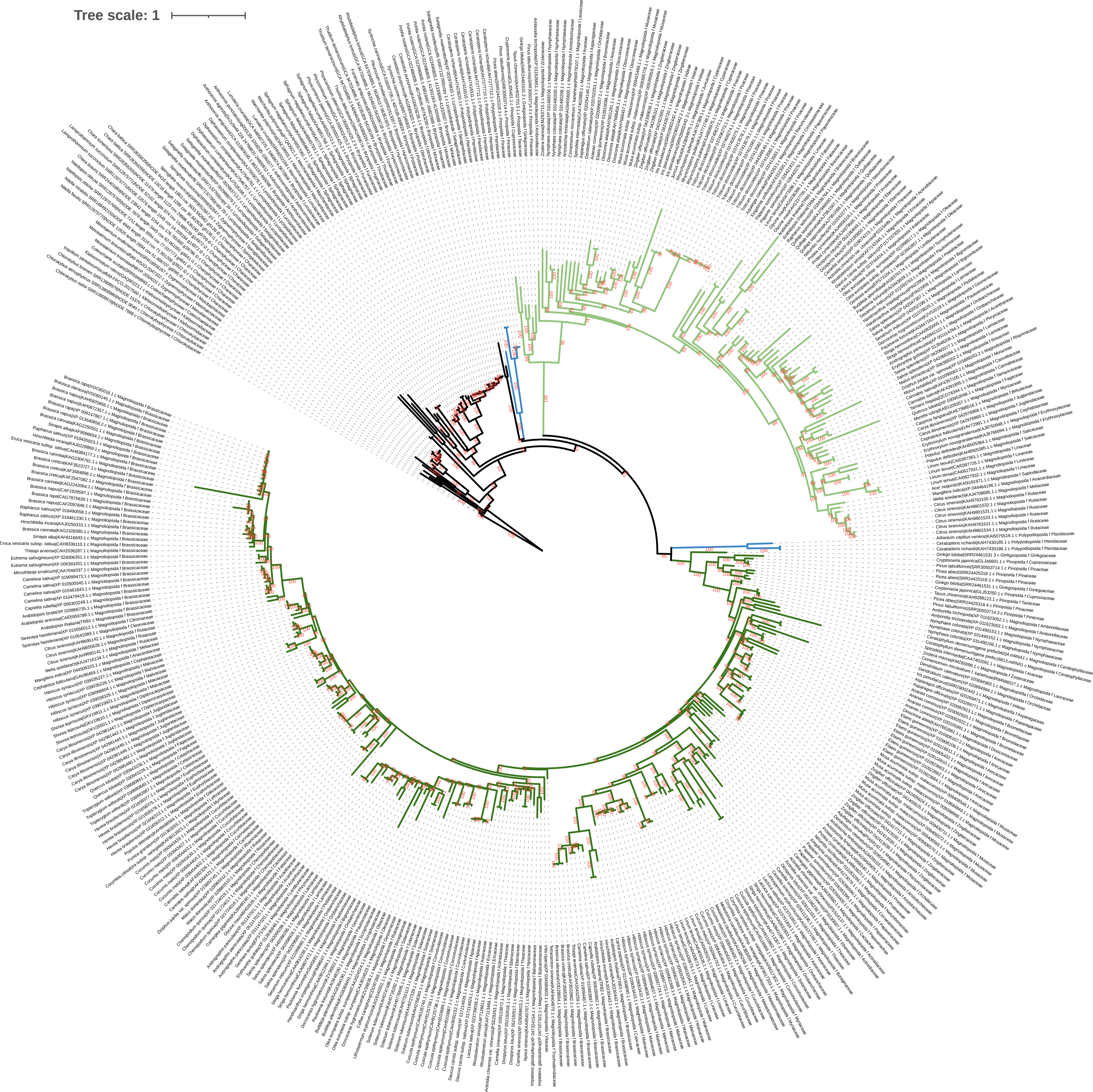
Tree scale: 1

Supplementary Figure 17. Phylogenetic analysis of TRB proteins across streptophyte taxa. Maximum likelihood (ML) phylogenetic trees were constructed to examine the evolutionary relationships of TRB proteins from various species, with accession numbers indicated for each sequence. Branch support was evaluated using ultrafast bootstrap approximation with 10,000 replicates; bootstrap values are shown below the branches. The trees were visualized using iTOL v6 (Letunic and Bork, 2024). Within Spermatophyta, TRB proteins segregate into two distinct clades, represented by light and dark green branches, highlighting their divergence in seed plants.

References

Letunic, I. and Bork, P. (2024) Interactive Tree of Life (iTOL) v6: recent updates to the phylogenetic tree display and annotation tool. *Nucleic Acids Res.*, **52**, W78–W82.

Nevosád, L., Klodová, B., Rudolf, J., Raček, T., Přerovská, T., Kusová, A., Svobodová, R., Honys, D. and Procházková Schrumpfová, P. (2025) GOLEM: A tool for visualizing the distribution of Gene regulatOry eLEMents within the plant promoters with a focus on male gametophyte. *Plant J.*, **121**, e70037.
